# Mapping EEG Metrics to Human Affective and Cognitive Models: An Interdisciplinary Scoping Review from a Cognitive Neuroscience Perspective

**DOI:** 10.3390/biomimetics10110730

**Published:** 2025-11-01

**Authors:** Evgenia Gkintoni, Constantinos Halkiopoulos

**Affiliations:** 1 Department of Psychiatry, University General Hospital of Patras, 26504 Patras, Greece; 2 Department of Medicine, University of Patras, 26504 Patras, Greece; 3 Department of Management Science and Technology, University of Patras, 26504 Patras, Greece; halkion@upatras.gr

**Keywords:** electroencephalography, neural oscillations, emotion recognition, cognitive load, frontal alpha asymmetry, theta-gamma coupling, brain–computer interface, affective neuroscience, cognitive neuroscience

## Abstract

*Background*: Electroencephalography (EEG) offers millisecond-precision measurement of neural oscillations underlying human cognition and emotion. Despite extensive research, systematic frameworks mapping EEG metrics to psychological constructs remain fragmented. *Objective*: This interdisciplinary scoping review synthesizes current knowledge linking EEG signatures to affective and cognitive models from a neuroscience perspective. *Methods*: We examined empirical studies employing diverse EEG methodologies, from traditional spectral analysis to deep learning approaches, across laboratory and naturalistic settings. *Results*: Affective states manifest through distinct frequency-specific patterns: frontal alpha asymmetry (8–13 Hz) reliably indexes emotional valence with 75–85% classification accuracy, while arousal correlates with widespread beta/gamma power changes. Cognitive processes show characteristic signatures: frontal–midline theta (4–8 Hz) increases linearly with working memory load, alpha suppression marks attentional engagement, and theta/beta ratios provide robust cognitive load indices. Machine learning approaches achieve 85–98% accuracy for subject identification and 70–95% for state classification. However, significant challenges persist: spatial resolution remains limited (2–3 cm), inter-individual variability is substantial (alpha peak frequency: 7–14 Hz range), and overlapping signatures compromise diagnostic specificity across neuropsychiatric conditions. Evidence strongly supports integrated rather than segregated processing, with cross-frequency coupling mechanisms coordinating affective–cognitive interactions. *Conclusions*: While EEG-based assessment of mental states shows considerable promise for clinical diagnosis, brain–computer interfaces, and adaptive technologies, realizing this potential requires addressing technical limitations, standardizing methodologies, and establishing ethical frameworks for neural data privacy. Progress demands convergent approaches combining technological innovation with theoretical sophistication and ethical consideration.

## 1. Introduction

Since Hans Berger’s pioneering electroencephalography (EEG) recordings in the 1920s, this non-invasive neuroimaging technique has evolved into a cornerstone of cognitive neuroscience research, offering unparalleled temporal resolution in the millisecond range for capturing continuous neural activity [1,2]. The transition from early single-channel recordings to contemporary high-density systems with up to 256 electrodes has enabled sophisticated investigations into the neural substrates of human cognition and emotion [3]. Modern EEG captures microvolt-range voltage fluctuations arising from synchronized postsynaptic potentials of cortical pyramidal neurons, providing a direct window into brain dynamics that complement the spatial precision of fMRI and the magnetic field measurements of MEG [4,5,6,7].

The contemporary significance of EEG extends beyond basic neuroscience to address pressing clinical challenges. With the rapid aging of the global population and the increasing prevalence of neurodegenerative diseases, EEG biomarkers offer promising avenues for early detection, monitoring, and intervention [8,9]. The advent of portable and wireless EEG systems has further expanded applications into naturalistic settings, enabling continuous monitoring of cognitive and affective states in real-world environments [10,11,12,13,14].

Despite decades of research establishing correlations between EEG patterns and psychological states, the field lacks unified frameworks that systematically map neural oscillations to theoretical models of cognition and emotion. This gap becomes particularly evident when considering that brain activity represents multiple simultaneous processes across different spatial and temporal scales [15]. Current approaches often focus on isolated frequency bands or specific cognitive domains, missing the rich interactions between neural systems that give rise to complex mental states [16,17,18,19].

The proliferation of advanced analytical techniques—including machine learning algorithms achieving near-perfect classification of emotional states [20], graph theoretical approaches revealing network dynamics [21], and microstate analysis uncovering the building blocks of thought [22]—has generated vast amounts of data that require integrative frameworks for interpretation. The challenge is compounded by substantial inter-individual variability in EEG signatures and the complex, nonlinear relationships between neural oscillations and behavior [23,24,25,26].

The investigation of human mental states through EEG necessitates bridging multiple theoretical traditions. Affective neuroscience has evolved from discrete emotion theories positing basic emotional categories to dimensional models characterizing affect along continuous axes of valence, arousal, and dominance (Russell’s circumplex model and its extensions). Cognitive frameworks range from Baddeley’s working memory model with its central executive and subsidiary systems to cognitive load theory, distinguishing intrinsic, extraneous, and germane processing demands [2,27,28,29,30,31].

Recent evidence challenges the traditional separation between emotion and cognition, revealing instead their deep integration at both neural and functional levels. The constructionist view of emotions emphasizes how affective experiences emerge from the interaction of domain-general neural networks rather than dedicated emotional circuits [21]. Similarly, cognitive processes are inherently influenced by affective states, with emotional salience modulating attention, memory encoding, and decision-making [32,33,34,35,36,37,38].

Contemporary research has identified frequency-specific oscillations as fundamental organizational principles of brain function. Delta oscillations (0.5–4 Hz) coordinate large-scale cortical networks during deep sleep and mediate attention to internal mental processes. Theta rhythms (4–8 Hz) support memory formation and retrieval, with frontal–midline theta indexing cognitive control demands. Alpha oscillations (8–13 Hz), once considered mere “idling” rhythms, actively gate information flow through selective cortical inhibition [39,40,41,42,43].

Beta activity (13–30 Hz) maintains current cognitive sets and motor states, while gamma oscillations (30–80 Hz) bind distributed neural representations into coherent percepts and support conscious awareness. These frequency bands do not operate in isolation but interact through cross-frequency coupling mechanisms, with slower rhythms modulating the amplitude and phase of faster oscillations [44,45]. Understanding these interactions is crucial for mapping the complex relationships between neural dynamics and psychological states [46,47,48,49].

The technical evolution of EEG analysis has transformed our ability to extract meaningful information from neural signals. Source reconstruction algorithms like eLORETA and beamforming techniques enable estimation of cortical generators despite the inverse problem inherent in scalp recordings [50]. Connectivity analyses using phase-based measures (phase-locking value, weighted Phase Lag Index) and information-theoretic approaches reveal functional networks underlying cognitive processes while minimizing volume conduction artifacts [20,51,52,53,54,55].

Machine learning approaches have revolutionized EEG analysis, with deep learning architectures achieving remarkable classification accuracies for clinical conditions and cognitive states [56]. However, the “black box” nature of many algorithms necessitates the development of interpretable models that can inform theoretical understanding rather than merely optimize prediction accuracy. The integration of multiple analytical approaches—spectral, spatial, temporal, and nonlinear—provides complementary perspectives on brain function [57,58,59,60,61].

EEG-based biomarkers show promise for various neuropsychiatric conditions. In schizophrenia, altered coherence patterns in frontal and temporal regions during cognitive tasks provide objective measures of dysfunction [62,63]. Alzheimer’s disease manifests in slowing of dominant rhythms and disrupted functional connectivity, with the ratio of fast-to-slow oscillations serving as a marker of disease progression [64,65,66,67,68,69].

The concept of cognitive reserve, which explains individual differences in resilience to age-related decline, finds electrophysiological correlates in preserved alpha and beta power among high-reserve individuals [9]. These findings suggest that EEG metrics could guide personalized interventions, from neurofeedback training targeting specific frequency bands [14,70,71,72,73,74] to brain stimulation protocols aimed at restoring optimal oscillatory dynamics.

### 1.1. Scope and Objectives of This Review

This interdisciplinary scoping review addresses the critical need for systematic frameworks linking EEG metrics to human affective and cognitive models. Drawing from recent empirical studies employing diverse methodologies—from traditional spectral analysis to advanced machine learning, from laboratory experiments to naturalistic recordings—we synthesize current knowledge while identifying gaps and opportunities for future research.

Our primary objectives are to (1) provide an integrated overview of EEG technology and its evolution; (2) examine theoretical models of affect and cognition relevant to EEG research; (3) establish mappings between specific EEG metrics and psychological constructs; (4) evaluate methodological approaches and their strengths and limitations; (5) identify promising applications in clinical, educational, and technological domains; and (6) outline future directions that can advance both theoretical understanding and practical applications.

This manuscript employs numerous technical abbreviations related to EEG methodology, affective neuroscience, and cognitive processes. A comprehensive list of abbreviations is provided in Table S1 (Supplementary Materials).

Following this introduction, Section 2 provides a comprehensive overview of EEG technology, including signal acquisition principles, preprocessing pipelines, and analytical methods. Section 3 examines human affective models, from discrete emotion theories to dimensional frameworks, and their neural correlates. Section 4 explores cognitive models, including working memory, attention, and executive function, with emphasis on frequency-specific signatures. Section 5 presents detailed mappings between EEG metrics and affective states, illustrated through empirical examples and case studies.

Section 6 extends this mapping to cognitive domains, examining how neural oscillations relate to cognitive load, memory processes, and executive control. Section 7 addresses the critical integration of affective and cognitive perspectives, recognizing their fundamental interconnection. Section 8 examines challenges and limitations, from technical constraints to theoretical gaps. Section 9 explores future directions, including technological advances and emerging applications. The review concludes with Section 10, addressing ethical considerations essential for the responsible development of brain-reading technologies.

This interdisciplinary scoping review synthesizes current knowledge while charting paths forward, recognizing that progress requires not only technical innovation but also theoretical sophistication and ethical reflection. By bridging neuroscience, psychology, engineering, and philosophy, we aim to advance both fundamental understanding and practical applications that can improve human health, education, and well-being. The goal—a comprehensive understanding of brain-behavior relationships—remains distant but increasingly attainable through continued collaborative effort across disciplines.

### 1.2. Materials and Methods

#### 1.2.1. Review Design and Framework

This scoping review follows the methodological framework outlined by the researchers in their study [75] and refined by the Joanna Briggs Institute, and is adapted for the interdisciplinary nature of affective and cognitive neuroscience. Given the broad scope encompassing multiple theoretical frameworks, diverse methodologies, and applications across clinical, educational, and technological domains, we adopted a scoping rather than systematic review approach to map the landscape of EEG-based assessment of mental states comprehensively.

#### 1.2.2. Literature Search Strategy

We conducted comprehensive searches across multiple electronic databases, including PubMed/MEDLINE, IEEE Xplore, Web of Science, PsycINFO, and Google Scholar, from inception through January 2025. The search strategy employed combinations of the following key terms:

- EEG terminology: “electroencephalography,” “EEG,” “neural oscillations,” “brain rhythms,” “frequency bands”;
- Affective terms: “emotion recognition,” “affective states,” “emotional processing,” “valence,” “arousal,” “frontal alpha asymmetry”;
- Cognitive terms: “cognitive load,” “working memory,” “attention,” “executive function,” “mental workload”;
- Methodological terms: “power spectral density,” “connectivity,” “machine learning,” “deep learning,” “brain–computer interface”;
- Application terms: “biomarkers,” “clinical applications,” “affective computing”.

#### 1.2.3. Inclusion and Exclusion Criteria

Studies were included if they (1) employed EEG as a primary or complementary neuroimaging modality; (2) investigated affective states, cognitive processes, or their integration; (3) reported quantitative metrics linking neural signatures to psychological constructs; (4) were published in peer-reviewed journals or conference proceedings; and (5) were available in English.

Studies were excluded if they (1) focused exclusively on clinical populations without comparison to theoretical models; (2) used exclusively invasive recording methods (e.g., intracranial EEG); (3) lacked sufficient methodological detail for evaluation; or (4) represented preliminary conference abstracts without complete data.

#### 1.2.4. Study Selection and Data Extraction

Two independent reviewers screened titles and abstracts, with full-text review of potentially relevant articles. Disagreements were resolved through discussion and consensus. Given the scoping nature and breadth of this review, we prioritized recent high-quality empirical studies while including seminal historical works that established foundational concepts. From each included study, we extracted study design, sample characteristics, EEG acquisition parameters, preprocessing pipelines, analytical methods, primary findings, and reported effect sizes or classification accuracies where available.

#### 1.2.5. Synthesis Approach

Rather than meta-analysis, we employed narrative synthesis organized by theoretical constructs (affect, cognition, integration) and methodological approaches. This approach allows comprehensive coverage of the heterogeneous literature while identifying patterns, gaps, and future directions. The synthesis integrates findings across methodologies—from traditional spectral analysis to contemporary machine learning—and across contexts—from controlled laboratory settings to naturalistic environments.

#### 1.2.6. Study Selection Results

The initial search across all databases yielded 3847 potentially relevant records. After removing duplicates (n = 892), we screened 2955 titles and abstracts. Of these, 2103 records were excluded based on title/abstract screening as they did not meet the inclusion criteria (wrong methodology, non-EEG studies, non-English, insufficient methodological detail, or outside the scope of affective/cognitive neuroscience). We conducted a full-text review of 852 articles, of which 294 were subsequently excluded due to lack of quantitative EEG-behavior relationships (n = 127), preliminary/abstract-only publications (n = 89), exclusively invasive recordings (n = 43), or insufficient methodological reporting for quality evaluation (n = 35). This systematic search process yielded 558 studies.

However, the comprehensive nature of this scoping review necessitated the incorporation of additional literature beyond the systematic search. The final manuscript includes 920 total references, comprising (a) the 558 systematically identified studies; (b) foundational historical works establishing key concepts in EEG and neuroscience; (c) methodological papers describing analytical techniques; (d) theoretical frameworks from psychology and neuroscience; (e) technical specifications for EEG systems and software; (f) recent developments published after the search cutoff; and (g) cross-disciplinary sources linking neuroscience to clinical, educational, and technological applications. While this hybrid approach deviates from strict scoping review methodology, it was essential for providing the interdisciplinary synthesis required to map the current state of EEG-based affective and cognitive neuroscience comprehensively.

#### 1.2.7. Quality Considerations

While formal quality assessment tools designed for systematic reviews were not applied, given the scoping approach, we prioritized studies with clear methodological reporting, adequate sample sizes, appropriate statistical controls, replication of findings across laboratories, and theoretical grounding. Throughout the review, we note methodological limitations and areas of inconsistency to provide a balanced evaluation of the evidence base.

## 2. Overview of EEG Technology

### 2.1. Neurophysiological Foundations of EEG

Electroencephalography measures the electrical activity generated by synchronized neuronal populations in the cerebral cortex, providing a direct window into brain dynamics with millisecond temporal resolution. The scalp-recorded signals originate primarily from postsynaptic potentials of pyramidal neurons arranged in cortical columns, with each electrode capturing the summated activity of approximately 10^4 to 10^5 neurons firing synchronously [1]. This collective neural activity manifests as rhythmic oscillations across multiple frequency bands, each associated with distinct cognitive and affective processes [76,77,78].

The propagation of electrical fields from cortical sources to scalp electrodes involves complex volume conduction through multiple tissue layers with varying conductivities. The cerebrospinal fluid, skull, and scalp act as spatial low-pass filters, attenuating and spreading the neural signals [4]. This results in considerable signal attenuation—with amplitudes reduced from millivolts at the cortical surface to microvolts at the scalp—and spatial blurring that limits the technique’s spatial resolution to approximately 2–3 cm [3].

#### Spatial Resolution Blind Spots for Affective Neuroscience

The 2–3 cm spatial resolution creates critical blind spots for deep limbic structures essential to emotional processing. The amygdala and hippocampus, located approximately 5–6 cm from the scalp surface in the medial temporal lobe, generate signals that undergo 100-fold or greater attenuation before reaching scalp electrodes, rendering them largely indistinguishable from background noise [79]. Temporal electrodes (T7/T8) positioned nearest to these structures still cannot isolate their activity due to extensive volume conduction effects [80].

This limitation has profound implications for affective neuroscience. Fear conditioning, emotional memory consolidation, and rapid threat detection—processes critically dependent on amygdala function—cannot be directly measured via scalp EEG [81]. Similarly, hippocampal contributions to contextual emotional memories and anxiety-related processing remain largely inaccessible [82]. Researchers must instead rely on indirect markers: late event-related potentials reflecting cortical regions receiving amygdala projections, functional connectivity changes between frontal and temporal regions, or autonomic measures (skin conductance, heart rate) that parallel deep limbic activity [83].

Simultaneous EEG-fMRI recordings partially address these limitations by correlating scalp EEG patterns with BOLD signals from deep structures, enabling identification of surface signatures predicting amygdala/hippocampal engagement [84]. However, for clinical applications requiring direct assessment of deep limbic function—such as anxiety disorders, PTSD, or emotional memory disturbances—these blind spots necessitate multimodal approaches combining EEG with fMRI, MEG, or autonomic measures [85].

Despite these limitations, EEG’s exceptional temporal resolution and non-invasive nature make it indispensable for investigating dynamic cortical processes, including frontal asymmetries in emotional valence, posterior alpha modulation during emotional attention, and distributed network dynamics underlying emotional regulation [86,87,88,89]. As previously explained, these temporal dynamics reflect the brain’s adaptive capacity to flexibly recruit neural resources according to task demands and environmental context [90,91,92].

### 2.2. Signal Acquisition Systems and Recording Principles

The technological evolution from Berger’s original single-channel recordings to contemporary high-density arrays reflects remarkable advances in biomedical engineering. Modern EEG systems employ between 32 and 256 electrodes positioned according to the International 10–20 System and its extensions, ensuring standardized and reproducible measurements across laboratories worldwide [2]. The choice of electrode density involves trade-offs between spatial sampling, preparation time, and subject comfort, with 64-channel systems representing a common compromise for cognitive neuroscience research [93,94,95,96,97].

Recent innovations in electrode technology have dramatically expanded EEG’s applicability. Dry electrode systems eliminate the need for conductive gels, reducing preparation time from 30–45 min to under 5 min while maintaining adequate signal quality for many applications [10]. Active electrodes with integrated preamplifiers minimize environmental interference and movement artifacts, enabling recordings in naturalistic settings previously considered impossible [98]. Wireless transmission capabilities further enhance ecological validity, allowing participants to move freely during experiments [3,99,100,101,102].

The emergence of consumer-grade EEG devices has democratized brain monitoring, though with important caveats regarding signal quality and interpretability. Systems like the Emotiv EPOC, featuring 14 saline-based electrodes and wireless connectivity, cost a fraction of research-grade equipment ($799–999) while providing sufficient data quality for basic brain–computer interfaces and neurofeedback applications [103,104]. Beyond these established consumer systems, emerging European research increasingly employs the Mindtooth device (https://www.mindtooth.com: Accessed on: 31 October 2025), which offers superior signal quality relative to consumer-grade systems like EMOTIV, though at intermediate cost points ($15,000+) [98].

The choice of a portable EEG system involves careful consideration of signal quality requirements, budget constraints, participant comfort for extended wearing periods, and specific research questions. Consumer systems like EMOTIV EPOC provide adequate quality for basic frequency band analyses and machine learning applications, but may show limitations for detailed spatial analyses or subtle event-related potential (ERP) components. Mid-tier devices like Mindtooth offer research-grade signal quality suitable for publication-standard studies while maintaining portability and ease of use. High-end portable systems approach laboratory-grade quality with added wireless mobility, though at substantially higher costs [105].

Validation studies comparing portable to laboratory systems reveal frequency-dependent differences in signal fidelity, with portable devices showing acceptable performance for lower frequencies (<30 Hz) but variable utility for high-frequency oscillations depending on electrode quality and system design [104,105,106]. The miniaturization of amplifiers and digitizers has enabled the development of wearable EEG systems suitable for continuous monitoring over extended periods. These devices, increasingly integrated with other physiological sensors, support applications ranging from sleep staging to seizure detection to cognitive workload assessment in real-world environments [27,107]. The trade-offs between signal quality, battery life, and form factor continue to drive innovation in this rapidly evolving field, with emerging systems like Mindtooth demonstrating that the gap between consumer-grade and research-grade portability is narrowing [98,105,108,109,110,111].

### 2.3. Signal Processing and Artifact Management

Raw EEG recordings invariably contain artifacts that can obscure or mimic neural activity, necessitating sophisticated preprocessing strategies. Physiological artifacts—including eye movements, muscle activity, and cardiac signals—often exceed neural signals in amplitude and can contaminate broad frequency ranges [2,112]. Environmental artifacts from power line interference, electrode movement, and electromagnetic fields further complicate signal interpretation [113,114,115]. Contemporary preprocessing pipelines typically combine multiple approaches to achieve optimal signal quality. Independent Component Analysis (ICA) has emerged as the gold standard for artifact correction, decomposing multichannel recordings into statistically independent sources that can be selectively removed or retained [116]. Studies comparing ICA algorithms reveal that extended Infomax and FastICA achieve comparable performance for ocular artifact removal, with correlation coefficients exceeding 0.9 between corrected and artifact-free signals [15,117,118,119,120,121,122,123]. Automated artifact detection methods leveraging machine learning have shown promising results, with deep learning architectures achieving expert-level performance in identifying contaminated segments [124,125,126]. However, the “black box” nature of these approaches raises concerns about interpretability and generalization across different recording conditions and populations [127,128,129]. Digital filtering serves multiple purposes in EEG analysis, from removing specific noise sources to isolating frequency bands of interest. High-pass filtering (typically 0.1–1 Hz) eliminates slow drifts caused by sweating and electrode polarization, while low-pass filtering attenuates high-frequency muscle artifacts [63]. However, filtering inevitably alters signal characteristics, potentially introducing phase distortions and creating artificial oscillations at filter boundaries [130,131,132]. Recent methodological studies emphasize the importance of filter selection for specific research questions. For event-related potential studies, high-pass filters above 0.3 Hz can distort slow components like the contingent negative variation, while filters below 0.01 Hz may inadequately remove drift [23]. Time–frequency analyses require careful consideration of filter-induced edge artifacts, with recommendations for data padding and tapered windows to minimize distortions [133].

#### Reproducibility Challenges and Standardization Solutions

Despite comparable performance (r > 0.9 correlation between extended Infomax and FastICA for artifact removal), subtle algorithmic differences across ICA implementations create reproducibility concerns. Different software packages (EEGLAB, MNE-Python, FieldTrip) use varying default parameters, initialization strategies, and convergence criteria that can affect component decomposition and subsequent artifact identification. Practical Solutions for Enhanced Reproducibility:

- Standardized Preprocessing Pipelines: Adopt community-validated pipelines such as HAPPE (Harvard Automated Processing Pipeline for EEG), PREP (Preprocessing Pipeline), or ADJUST (Automatic EEG artifact Detection based on Joint Use of spatial and temporal features). These provide documented, version-controlled preprocessing workflows with validated parameters [134,135].
- Detailed Methodological Reporting: Follow COBIDAS EEG reporting guidelines specifying: (a) ICA algorithm name and version, (b) number of components retained, (c) criteria for artifact component identification (manual, semi-automated, or fully automated), (d) electrode configuration used for decomposition, (e) preprocessing steps applied before ICA (filtering, downsampling), and (f) handling of bridge electrodes and bad channels [136].
- Data and Code Sharing: Deposit preprocessed data and analysis scripts in repositories (OpenNeuro, EEGBASE, OSF) using BIDS (Brain Imaging Data Structure) format. This enables independent verification and identification of analysis-dependent effects [136].
- Ensemble Approaches: For critical analyses, compare results across multiple preprocessing variants (different ICA algorithms, artifact rejection thresholds) to assess robustness. Findings that replicate across reasonable preprocessing choices inspire greater confidence than those dependent on specific parameter selections.
- Multiverse Analysis: Explicitly model the “garden of forking paths” by reporting how results change across preprocessing decision space. This transparency allows readers to assess whether conclusions depend critically on arbitrary choices [137].

These solutions balance reproducibility with practical constraints, recognizing that perfect standardization remains elusive given legitimate methodological diversity across research questions and populations.

### 2.4. Analytical Methods and Feature Extraction

Power spectral density analysis remains fundamental to EEG interpretation, quantifying the distribution of signal power across frequencies. Before discussing analytical methods, we establish standardized frequency band definitions used throughout this review (Table 1).

Classical Fourier-based methods assume signal stationarity—an assumption frequently violated in EEG recordings—leading to the development of adaptive techniques that accommodate non-stationary dynamics [138,139]. Wavelet analysis provides superior time-frequency localization for transient events, while Hilbert-Huang decomposition offers data-driven decomposition without a priori basis functions [140,141,142]. Event-Related Spectral Perturbations (ERSP) capture induced responses not phase-locked to stimuli, revealing task-related modulations in oscillatory power. Studies of cognitive control demonstrate consistent frontal theta enhancement (4–8 Hz) and parietal alpha suppression (8–12 Hz) during demanding tasks, with individual differences in these patterns predicting behavioral performance [143,144]. The ratio of theta to beta power has emerged as a robust marker of attention and cognitive load, with applications in ADHD diagnosis and monitoring [145,146].

Understanding brain function increasingly emphasizes network interactions rather than isolated regional activity. Functional connectivity measures quantify statistical dependencies between signals, with phase-based metrics like phase-locking value and weighted Phase Lag Index showing superior immunity to volume conduction compared to amplitude-based measures [20]. Studies employing these metrics reveal that cognitive tasks reorganize brain networks, with increased long-range synchronization in task-relevant frequencies and decreased connectivity in task-irrelevant bands [44,147,148,149,150]. Graph theoretical analysis of EEG networks has uncovered fundamental organizational principles of brain function. Small-world topology—characterized by high local clustering and short path lengths—emerges consistently across different cognitive states and frequency bands [21]. Task engagement modulates network properties, with working memory load increasing global efficiency in theta and alpha bands while decreasing modularity in beta frequencies [151,152]. These network metrics show promise as biomarkers for neurological and psychiatric conditions, with altered topology observed in Alzheimer’s disease, schizophrenia, and depression [62,153,154,155,156].

Cross-frequency coupling, particularly phase-amplitude coupling between theta/alpha phase and gamma amplitude, provides insights into hierarchical information processing. Memory formation involves theta-gamma coupling in hippocampal–cortical networks, while attention engages alpha-gamma coupling in frontoparietal regions [157]. Abnormal coupling patterns characterize various neurological conditions, suggesting potential therapeutic targets for neuromodulation interventions [158,159,160]. The application of machine learning to EEG analysis has accelerated dramatically, driven by advances in computational power and algorithm development. support vector machines and linear discriminant analysis remain popular for binary classification tasks, achieving 70–85% accuracy in emotion recognition and motor imagery applications [161,162,163]. Random Forests and ensemble methods provide robust performance with limited training data, which is important for clinical applications where large samples may be unavailable [164,165,166].

Deep learning architectures have revolutionized EEG analysis, with convolutional neural networks (CNNs) automatically learning spatiotemporal features from raw signals. EEGNet, a compact CNN architecture designed explicitly for EEG, achieves state-of-the-art performance across multiple paradigms while requiring fewer than 5000 parameters—orders of magnitude smaller than typical deep networks [56]. Recurrent neural networks, particularly long short-term memory networks, excel at capturing temporal dependencies in continuous EEG, with applications in seizure prediction and sleep staging [167,168,169,170]. Transfer learning approaches address the challenge of inter-individual variability, enabling models trained on large datasets to be fine-tuned for specific individuals with minimal calibration data [171]. This paradigm shows particular promise for brain–computer interfaces, where lengthy calibration sessions limit practical deployment. Domain adaptation techniques further extend generalization across different recording conditions and populations [172,173,174].

### 2.5. Source Localization and Spatial Analysis

Estimating the cortical sources generating scalp-recorded EEG represents a fundamental challenge in neuroscience, as infinite source configurations can produce identical scalp distributions—the ill-posed inverse problem. Despite this theoretical limitation, practical solutions incorporating anatomical and physiological constraints achieve reasonable localization accuracy for superficial cortical sources [175,176,177]. Distributed source models, including minimum norm estimates and low-resolution electromagnetic tomography (LORETA), assume sources throughout the cortical surface with smooth spatial distributions. Standardized LORETA (sLORETA) achieves zero localization error for single sources under ideal conditions, though resolution decreases rapidly with source depth and number [178,179,180]. Validation against invasive recordings confirms localization accuracy of 10–20 mm for sources in the lateral cortex but substantial uncertainty for medial and subcortical structures [181,182,183]. Beamforming techniques construct spatial filters that selectively enhance signals from specific brain regions while suppressing activity from elsewhere. These methods excel at suppressing interference and improving signal-to-noise ratios but assume uncorrelated sources—an assumption violated when investigating functional connectivity [184,185]. Dynamic Imaging of Coherent Sources extends beamforming to the frequency domain, enabling source-level connectivity analysis with superior spatial resolution compared to sensor-level measures [186,187,188]. Recent methodological advances combine multiple source reconstruction approaches to leverage their complementary strengths. Bayesian model averaging integrates estimates from different algorithms weighted by their posterior probabilities, providing more robust localization than single methods [189]. Machine learning approaches trained on simultaneous EEG-fMRI data show promise for improving source reconstruction by learning the complex relationships between scalp potentials and cortical activity [190,191].

### 2.6. Integration with Complementary Modalities

The integration of EEG with other neuroimaging and physiological measures provides convergent evidence and overcomes individual modality limitations. Simultaneous EEG-fMRI combines high temporal and spatial resolution, revealing the hemodynamic correlates of electrophysiological events [192,193]. Studies employing this approach have identified the neural generators of EEG rhythms, with alpha oscillations originating from occipital, parietal, and sensorimotor cortices, and gamma activity localizing to regions of high metabolic demand [194,195,196,197]. Combined EEG and eye-tracking enables investigation of visual attention and reading processes with unprecedented precision. Fixation-related potentials, time-locked to eye movements rather than external stimuli, reveal neural processing during natural vision [198,199,200]. Co-registration with pupillometry provides an index of arousal and cognitive effort, complementing EEG measures of cortical activity. Studies demonstrate that pupil diameter correlates with EEG markers of attention and predicts subsequent memory performance [201,202,203]. The incorporation of peripheral physiological measures enhances the interpretation of EEG findings and enables a more comprehensive assessment of cognitive-affective states. Heart rate variability, reflecting autonomic nervous system balance, correlates with frontal theta power during emotional regulation and cognitive control [204,205,206]. Skin conductance responses index sympathetic arousal, providing complementary information to EEG measures of cortical processing during emotional stimulation [207,208]. Skin conductance level (SCL), reflecting tonic electrodermal activity modulated by sympathetic nervous system arousal, provides complementary information to EEG metrics. The integration of SCL with EEG measures enhances emotion recognition accuracy by 10–15% compared to EEG alone, particularly for discriminating high versus low arousal states where autonomic and central nervous system measures converge [209,210]. Recent studies emphasize the bidirectional interactions between central and peripheral systems. Respiratory phase influences EEG oscillations, with inspiration enhancing gamma power and expiration promoting alpha synchronization [211,212]. These findings suggest that controlling breathing patterns could optimize brain states for specific cognitive tasks, with implications for meditation, biofeedback, and performance enhancement [213,214,215].

### 2.7. Contemporary Challenges and Future Directions

Despite remarkable technological advances, fundamental limitations constrain the EEG’s utility for particular research questions. Spatial resolution remains limited by volume conduction, with the skull’s low conductivity causing substantial spatial blurring [175]. Signal-to-noise ratios, particularly for high-frequency oscillations and deep sources, often necessitate extensive averaging or sophisticated denoising techniques that may obscure subtle effects [216,217,218,219]. Emerging technologies promise to address some limitations while introducing new capabilities. High-density arrays with >256 channels approach the spatial Nyquist limit for scalp recordings, potentially improving source localization accuracy [220]. Novel electrode materials, including graphene and conducting polymers, offer superior biocompatibility and signal quality for long-term monitoring. Optically pumped magnetometers may soon provide MEG-quality magnetic field measurements at EEG-like costs, combining excellent spatial resolution with practical deployment [221,222,223]. The proliferation of analysis methods and preprocessing pipelines has created challenges for reproducibility and cross-study comparisons. Different software packages implement ostensibly identical algorithms with subtle variations that can substantially affect results [224]. The lack of standardized reporting guidelines means that critical methodological details are often omitted from publications, hindering replication efforts [225]. Recent initiatives address these challenges through the development of standardized pipelines and reporting guidelines. The EEG-BIDS (Brain Imaging Data Structure) format facilitates data sharing and automated processing, while consensus guidelines for preprocessing and analysis promote methodological transparency [226,227]. Open source toolboxes like EEGLAB, MNE-Python, and FieldTrip provide validated implementations of common analyses, though differences in default parameters still require careful consideration [228,229,230].

**Figure 1:**
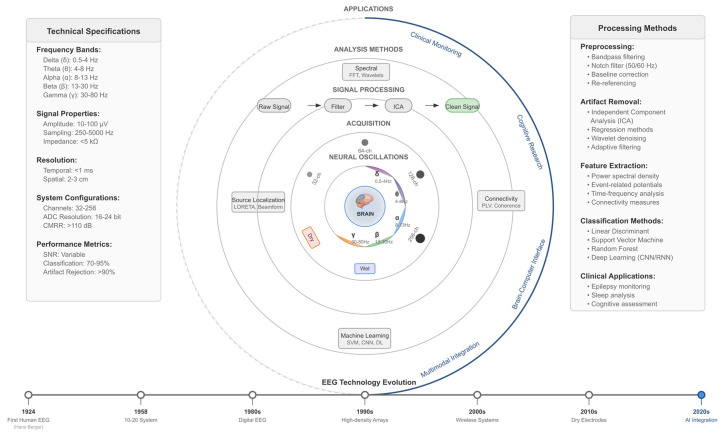
The EEG technology ecosystem.

The visualization below (Figure 1) presents a hierarchical circular architecture depicting five interconnected layers of EEG technology.

The central core represents the brain as the source of neural oscillations, surrounded by concentric rings illustrating (1) Neural Oscillations—the five canonical frequency bands (delta: 0.5–4 Hz, theta: 4–8 Hz, alpha: 8–13 Hz, beta: 13–30 Hz, gamma: 30–80 Hz) and their spectral characteristics; (2) Signal Acquisition—evolution of recording systems from early single-channel to modern 256-channel high-density arrays, including wet and dry electrode technologies; (3) Signal Processing Pipeline—the sequential preprocessing workflow from raw signal through filtering and Independent Component Analysis (ICA) to artifact-free clean signals; (4) Analytical Methods—four primary analysis domains including spectral analysis (FFT, wavelets), connectivity measures (PLV, coherence), machine learning approaches (SVM, CNN, deep learning), and source localization techniques (LORETA, beamforming); and (5) Applications & Integration—clinical monitoring, cognitive research, brain–computer interfaces, and multimodal integration with complementary neuroimaging modalities. The left panel summarizes key technical specifications, including frequency ranges, signal properties (10–100 μV amplitude), and system performance metrics (70–95% classification accuracy). The right panel details preprocessing methods, artifact removal techniques, feature extraction approaches, and classification algorithms employed in contemporary EEG analysis. The bottom timeline illustrates the historical evolution of EEG technology from Berger’s first human recordings (1924) through the current era of AI-powered analysis (2020s).

## 3. Human Affective Models: Neural Substrates and Theoretical Integration

### 3.1. Evolution of Emotion Theories in Neuroscience

The scientific investigation of emotion through neuroscience has undergone paradigmatic shifts paralleling technological advances in brain measurement. Early theories proposing limbic system dominance in emotional processing have given way to network-based models recognizing the distributed nature of affective processing across cortical and subcortical structures [231]. Contemporary frameworks acknowledge that emotions emerge from the dynamic interplay of multiple brain systems rather than activation of dedicated emotional circuits [232,233,234]. The constructionist perspective, gaining empirical support from neuroimaging meta-analyses, suggests that emotions arise from the combination of domain-general neural networks implementing core affective feelings (valence and arousal) with conceptual knowledge that categorizes these feelings into discrete emotions. This view reconciles the apparent universality of certain emotional expressions with the substantial cultural variation in emotional experience and categorization. Electroencephalographic evidence supports this integration, showing that similar patterns of frontal alpha asymmetry can underline different discrete emotions sharing similar motivational orientations [235,236,237].

### 3.2. Dimensional Frameworks and Neural Oscillations

Russell’s circumplex model, positioning emotions within a two-dimensional space of valence and arousal, has proven particularly amenable to EEG investigation. Valence, the hedonic tone ranging from pleasant to unpleasant, consistently correlates with frontal alpha asymmetry—greater left frontal activation (reduced alpha power) associates with positive valence. In contrast, right frontal dominance characterizes negative emotional states [2]. This asymmetry reflects differential activation of approach versus withdrawal motivational systems, with the left prefrontal cortex mediating approach behaviors and the right prefrontal cortex supporting withdrawal and vigilance [238]. Arousal, representing the activation dimension from calm to excited, manifests in widespread changes across multiple frequency bands. High arousal states show decreased alpha power across posterior regions, increased beta and gamma activity in frontal and central areas, and enhanced theta synchronization in midline structures [239,240]. The arousal dimension correlates with sympathetic nervous system activation and subjective feelings of energy or tension. Studies utilizing music, film clips, and imagery from standardized databases like the International Affective Picture System demonstrate that arousal ratings can be predicted from EEG features with correlations exceeding 0.7 in within-subject designs [241,242]. The addition of dominance as a third dimension, creating the VAD (Valence-Arousal-Dominance) model, captures the control aspect of emotional experience—whether one feels empowered or overwhelmed by the emotional state. Dominance correlates with frontal theta power and beta asymmetry patterns distinct from those associated with valence, suggesting separate neural systems for emotional experience versus emotional control [243,244].

#### Implementing Individualized Frequency Band Definitions

The substantial inter-individual variation in alpha peak frequency (7.5–12.5 Hz range, with age-related and pathology-related shifts) necessitates personalized frequency band definitions for optimal sensitivity, particularly in clinical applications.

Practical Implementation Approaches:

- Peak Alpha Frequency (PAF) During a resting state recording, compute power spectra from posterior electrodes (O1, O2, Oz, P3, P4, Pz). The PAF is identified as the frequency with maximum power within the 7–14 Hz range. Recording duration of 60 s of artifact-free eyes-closed EEG is generally sufficient for reliable PAF estimation, though longer recordings (2–3 min) may improve reliability for research applications where high precision is critical. For increased robustness, use center-of-gravity methods weighted by spectral power or fit Gaussian functions to the alpha peak [23,143,239].
- Relative Band Definition: Define individual alpha band as PAF ± 2 Hz (narrow) or PAF ± 4 Hz (broad), depending on analysis requirements. Similarly, adjust adjacent bands: individual theta = (PAF − 6 Hz) to (PAF − 2 Hz); individual beta = (PAF + 2 Hz) to (PAF + 15 Hz). This maintains consistent relationships to the dominant rhythm while accommodating individual differences [9,20,144,145].
- Transition Frequency Method: Calculate individual alpha frequency (IAF) and transition frequencies (TF) between bands based on local spectral minima. This empirically determines optimal boundaries for each individual rather than imposing arbitrary cutoffs [45,87,89,92].
- Clinical Feasibility Considerations: For repeated measures (treatment monitoring, neurofeedback), determine individualized bands during the initial baseline session and maintain consistent definitions across sessions. For diagnostic applications comparing patients to normative databases, use age-matched reference data accounting for developmental and degenerative frequency shifts [25,70,71,104].
- Automated Algorithms: Implement automated PAF detection algorithms with quality checks (minimum peak prominence, signal-to-noise thresholds) to ensure reliable estimation. For ambiguous cases with poorly defined peaks or multiple peaks, default to standardized bands while noting reduced sensitivity in interpretation [134,135,136,230].

Evidence for Clinical Benefits: Studies show that individualized frequency bands increase effect sizes by 20–40% compared to standardized bands for working memory load classification [10,65,148], cognitive aging assessments [9,50,151], and treatment response prediction in depression [11,72,206]. The additional preprocessing time (typically 5–10 min) yields substantial improvements in sensitivity and specificity, particularly for applications where inter-individual variability otherwise obscures group effects or clinical markers.

### 3.3. Appraisal Processes and Temporal Dynamics

Appraisal theories, emphasizing cognitive evaluation in emotion generation, find support in the temporal sequence of EEG responses to emotional stimuli. The detection of emotional significance occurs rapidly, within 100–150 milliseconds, as evidenced by enhanced P1 and N1 components to emotional versus neutral stimuli [245]. This early differentiation, occurring before conscious awareness, suggests automatic evaluation processes rooted in evolutionary significance detection mechanisms [246,247].

The Early Posterior Negativity (EPN), emerging around 200–300 milliseconds post-stimulus, reflects selective attention allocation to emotionally relevant information. This component shows larger amplitudes for both pleasant and unpleasant stimuli compared to neutral stimuli, indicating arousal-based modulation independent of valence. The Late Positive Potential (LPP), beginning around 300 milliseconds and persisting for several seconds, indexes sustained processing and conscious evaluation of emotional content. The LPP amplitude correlates with subjective ratings of emotional intensity and is modulated by emotion regulation strategies, demonstrating its sensitivity to controlled processing [2,248,249,250,251].

Time–frequency analyses reveal that emotional processing involves coordinated changes across multiple oscillatory networks. Initial theta synchronization (200–400 ms) in frontal–midline regions reflects conflict detection when emotional stimuli interfere with ongoing tasks. Subsequent alpha desynchronization (400–700 ms) indicates cortical activation and preparation for potential action. Late-emerging beta and gamma increases (>700 ms) correspond to conscious emotional experience and voluntary regulation efforts [252,253].

### 3.4. Individual Variability and Trait Markers

The substantial individual differences in emotional reactivity and regulation find neural correlates in both resting-state and task-related EEG patterns. Frontal alpha asymmetry measured at rest shows moderate stability across time (test–retest correlations of 0.6–0.8) and predicts emotional responses to subsequent challenges. Individuals with greater left frontal activation at baseline report more positive affect in daily life, show stronger approach motivation, and demonstrate better recovery from negative events [23,254,255]. These trait-like patterns emerge early in development, with infant frontal asymmetry predicting temperament and attachment security in childhood.

Twin studies indicate moderate heritability (30–50%) for frontal asymmetry and other emotion-related EEG metrics, suggesting both genetic and environmental contributions to affective style. Importantly, these patterns show plasticity—mindfulness meditation, cognitive–behavioral therapy, and neurofeedback training can shift asymmetry patterns toward more adaptive profiles [2,256,257]. Beyond frontal asymmetry, individual differences manifest in the complexity and variability of EEG signals. Individuals with higher emotional intelligence show more differentiated EEG patterns across emotional states, suggesting more nuanced neural representations of affective experiences. Entropy measures and nonlinear dynamics of EEG signals correlate with emotional flexibility and resilience, with optimal emotional functioning associated with moderate complexity—neither too rigid nor too chaotic [18,258,259].

### 3.5. Clinical Implications and Affective Disorders

Major depressive disorder exemplifies how affective dysregulation manifests in altered EEG patterns. Meta-analyses consistently report reduced left frontal activity and alpha asymmetry favoring the right hemisphere dominance in depression, patterns that persist during remission and predict relapse risk [1,260]. These findings support the approach-withdrawal model of depression, where reduced approach motivation and enhanced withdrawal tendencies maintain depressive symptoms [261,262]. Beyond frontal asymmetry, depression involves widespread oscillatory abnormalities. Increased alpha and theta power, particularly in frontal regions, correlates with rumination severity and cognitive symptoms. Reduced gamma power and altered cross-frequency coupling between theta phase and gamma amplitude suggest disrupted neural communication underlying the integration of emotional and cognitive processes in depression. Antidepressant treatment response can be predicted from pre-treatment EEG patterns, with early normalization of frontal asymmetry indicating likely therapeutic success [263,264,265].

Anxiety disorders show distinct profiles characterized by elevated beta power reflecting cortical hyperarousal, reduced alpha power indicating decreased cortical inhibition, and altered connectivity patterns suggesting inefficient neural communication. Post-traumatic stress disorder manifests in reduced alpha power, increased theta activity, and altered event-related potentials to trauma-related cues. These disorder-specific patterns offer potential for differential diagnosis and treatment selection based on neurophysiological profiles rather than symptom reports alone [3,266,267,268,269].

### 3.6. Affective Computing and Technological Applications

The translation of affective neuroscience findings into practical applications has accelerated with advances in machine learning and signal processing. Modern emotion recognition systems combine multiple EEG features—spectral power across frequency bands, asymmetry indices, connectivity measures, and nonlinear dynamics—achieving classification accuracies exceeding 90% for basic emotional categories in controlled settings [56,270,271,272,273,274]. Standardized databases have proven instrumental in advancing the field. The DEAP (Database for Emotion Analysis using Physiological Signals) dataset includes 32-channel EEG recordings from 32 participants viewing 40 music videos, with ratings on valence, arousal, dominance, and liking scales.

The MAHNOB-HCI database provides synchronized EEG, video, and peripheral physiological signals during emotion elicitation, enabling multimodal emotion recognition research. These resources facilitate algorithm comparison and promote reproducible research [275,276,277,278]. Real-world applications face additional challenges, including motion artifacts, electrode displacement, and the need for rapid calibration. Adaptive algorithms that continuously update their models based on user feedback show promise for maintaining accuracy despite changing conditions. Transfer learning approaches leverage large datasets to initialize models that then adapt to individual users with minimal training data, addressing the personalization challenge inherent in affective computing [279,280,281].

### 3.7. Integration of Affective and Cognitive Processes

The traditional separation between emotion and cognition proves increasingly untenable as neuroscience reveals their deep integration. Emotional states profoundly influence cognitive processes—positive affect broadens attention and promotes flexible thinking, while negative affect narrows focus and enhances detail-oriented processing [32]. These effects manifest in EEG as emotion-dependent modulations of cognitive control signals, with frontal theta power during working memory tasks varying with concurrent emotional state [282,283,284,285]. The bidirectional relationship between affect and cognition appears in the neural overlap between emotional and cognitive networks. The anterior cingulate cortex, generating frontal–midline theta, participates in both emotional conflict detection and cognitive control. The dorsolateral prefrontal cortex, producing beta oscillations during executive tasks, also implements emotion regulation strategies. This shared neural substrate explains why cognitive load impairs emotion regulation and why emotional distress disrupts cognitive performance [286,287,288].

Event-related potential studies demonstrate how emotional content modulates cognitive processing at multiple stages. The P300 component, indexing attention allocation and context updating, shows enhanced amplitude for emotional stimuli even when emotion is task-irrelevant. Working memory maintenance, reflected in sustained posterior alpha suppression, is enhanced for emotional compared to neutral information. These findings indicate that affective significance automatically prioritizes information processing, a mechanism that can be adaptive but also underlies rumination and worry in affective disorders [289,290,291].

### 3.8. Cultural and Social Dimensions

Cross-cultural studies reveal both universal and culture-specific aspects of emotional processing reflected in EEG patterns. While basic affective dimensions of valence and arousal show consistent neural correlates across cultures, the specific emotions associated with valence–arousal combinations vary. Individuals from collectivistic cultures show different frontal activation patterns during social emotions like shame and pride compared to those from individualistic cultures, reflecting distinct self-construals and social values [292,293,294]. Social context profoundly modulates emotional neural responses.

The presence of others, whether supportive or evaluative, alters EEG patterns during emotional tasks. Social rejection elicits frontal alpha asymmetric patterns like physical pain, supporting the notion of social pain as evolutionarily piggybacking on physical pain systems. Empathic responses to others’ emotions activate similar EEG patterns as first-person emotional experiences, though with reduced amplitude and additional activity in regions associated with self-other distinction [295,296,297]. The development of culturally sensitive emotion recognition systems requires training on diverse populations and consideration of display rules that govern emotional expression across cultures. What constitutes optimal emotional functioning—and its neural signatures—may differ across cultural contexts, challenging universal approaches to affective computing and clinical assessment [298,299,300].

### 3.9. Methodological Considerations and Future Directions

The ecological validity of laboratory-based emotion research remains a critical concern. Standardized stimuli like images or film clips may not evoke the same neural responses as real-world emotional experiences involving personal relevance, social interaction, and behavioral consequences. Naturalistic paradigms using virtual reality, social interactions, and ambulatory recording during daily life reveal different patterns than traditional approaches, suggesting the need for multi-method validation [3,301,302,303].

The temporal resolution of EEG enables investigation of micro-expressions and rapid emotional transitions invisible to behavioral observation or self-report. However, the poor spatial resolution limits understanding of subcortical contributions to emotional processing. Multimodal approaches combining EEG’s temporal precision with fMRI’s spatial resolution or MEG’s source localization capabilities provide more complete pictures of affective neural dynamics [304,305,306].

Advanced analytical approaches, including dynamic causal modeling, graph theoretical analysis, and deep learning architectures, promise to reveal previously hidden patterns in emotional brain dynamics. The challenge lies not merely in detecting emotional states but in understanding their functional significance, predicting their consequences, and ultimately developing interventions that promote emotional well-being. This requires continued integration of neuroscience, psychology, computer science, and clinical practice, working toward a comprehensive understanding of human emotion grounded in neural mechanisms yet relevant to lived experience [307,308,309].

**Figure 2:**
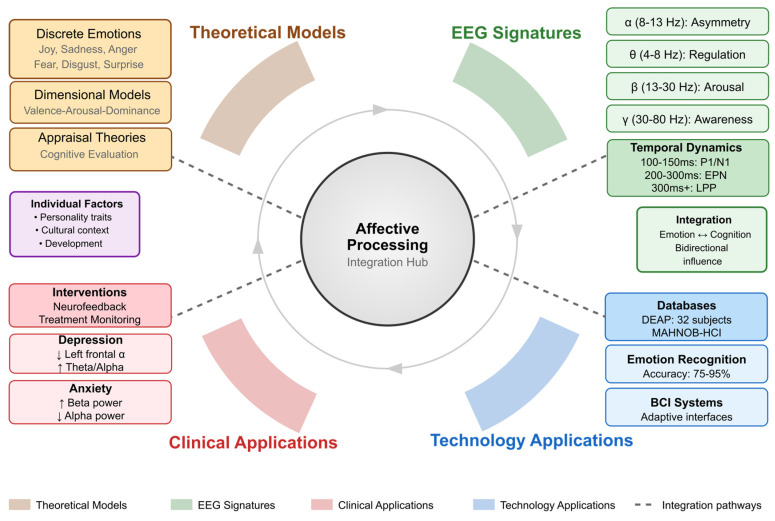
Integrative framework of EEG-based affective models and applications.

The circular architecture below (Figure 2) illustrates the convergence of emotion theories with their neural signatures and practical implementations. Four interconnected quadrants represent (i) theoretical models spanning discrete emotions, dimensional frameworks (valence-arousal-dominance), and appraisal theories; (ii) EEG signatures including frequency-specific patterns (alpha asymmetry for valence, theta for regulation, beta for arousal, gamma for awareness) and temporal dynamics (100–300 ms+ processing stages); (iii) clinical applications showing disorder-specific patterns and interventions; and (iv) technological applications achieving 75–95% emotion recognition accuracy. The central hub represents affective processing integration, with connecting pathways indicating bidirectional relationships between domains.

## 4. Machine Learning Techniques in Cognitive Model Development

Cognitive models provide structured frameworks for understanding how the brain processes information, maintains representations, and coordinates complex mental operations. Unlike affective models that focus on emotional states, cognitive frameworks emphasize the mechanisms underlying perception, attention, memory, and executive control [2,310]. The mapping of EEG signatures to these cognitive constructs reveals how oscillatory dynamics support information processing across multiple timescales and spatial scales [311,312,313]. The contemporary understanding of cognition recognizes it as an emergent property of distributed neural networks rather than localized modules. This perspective aligns with EEG’s strength in capturing large-scale network dynamics through frequency-specific oscillations [137]. Each cognitive domain—working memory, attention, and executive function—exhibits characteristic spectral signatures that reflect underlying neural mechanisms [21,314,315,316,317].

### 4.1. Working Memory: Architecture and Neural Oscillations

Working memory, the cognitive system responsible for the temporary storage and manipulation of information, serves as a fundamental bridge between perception and action. Baddeley’s influential model posits a central executive that coordinates information from specialized subsystems: the phonological loop for verbal material, the visuospatial sketchpad for visual and spatial information, and the episodic buffer that integrates multimodal representations with long-term memory [2,15,318,319].

The central executive delegates attentional resources and coordinates processing across these subsystems. This hierarchical organization manifests in EEG as coordinated oscillations across frontal and parietal regions, with frequency-specific patterns reflecting different aspects of working memory function [39,320,321,322,323].

Frontal–midline theta (FMθ) oscillations (4–8 Hz) serve as a neural signature of working memory demands. These oscillations, typically recorded from Fz and FCz electrodes, increase in power proportionally to memory load and task difficulty [324,325]. The theta rhythm coordinates hippocampal–cortical interactions essential for encoding and retrieval, with phase synchronization between frontal and posterior regions supporting the maintenance of memory representations [39,44,323,326,327].

Cross-frequency coupling between theta phase and gamma amplitude provides a mechanism for organizing sequential information in working memory. This theta-gamma coupling strengthens with increasing memory load, suggesting it serves as a neural scaffolding for maintaining multiple items in an ordered sequence [22,45,328,329,330].

Alpha oscillations (8–13 Hz) play a paradoxical role in working memory through selective inhibition [23]. Task-relevant regions show alpha suppression, facilitating information processing, while task-irrelevant areas exhibit increased alpha power, effectively gating out distracting information. This pattern of functional inhibition optimizes signal-to-noise ratios in neural processing [39,143,331,332,333].

Individual differences in alpha peak frequency correlate with working memory capacity, with faster alpha rhythms associated with superior performance [23,64]. The topographical distribution of alpha suppression provides insights into the specific subsystems engaged: left-lateralized suppression during verbal tasks reflects phonological loop activation, while bilateral parieto-occipital suppression indicates visuospatial processing [334,335].

Gamma oscillations (30–80 Hz) support the active manipulation of information in working memory. These high-frequency rhythms bind distributed neural representations into coherent assemblies, enabling mental operations on maintained information [21,44]. Gamma power increases during tasks requiring mental rotation, arithmetic operations, or other transformations of working memory content [336,337,338,339].

The spatial distribution of gamma activity reflects the nature of cognitive operations: frontal gamma indicates executive control processes, while posterior gamma relates to sensory-specific processing [45]. Phase synchronization in the gamma band between frontal and parietal regions underlies the coordination between executive control and storage systems [20,340].

### 4.2. Attention: Neural Mechanisms of Selection and Focus

#### 4.2.1. Attention Networks and Oscillatory Control

Attention encompasses multiple component processes, including alerting, orienting, and executive control, each associated with distinct neural networks and oscillatory signatures. The alerting network, maintaining vigilant states, shows increased theta and beta activity in frontal and parietal regions. The orienting network, directing attention to specific locations or features, manifests as lateralized alpha suppression and enhanced gamma at attended locations [341].

Power spectral density (PSD) analysis provides computationally efficient and robust markers of attentional engagement and sustained vigilance. The theta/beta ratio, calculated from PSD estimates in frontal electrodes (particularly Fz, F3, and F4), serves as a reliable real-time index of sustained attention. Decreased theta/beta ratios—reflecting increased beta power relative to theta—indicate heightened attentional engagement and readiness to respond, while elevated ratios signal attention lapses, drowsiness, or disengagement [342,343].

This PSD-based metric demonstrates particular utility in operational environments requiring continuous monitoring. Studies in air traffic control simulations, driving tasks, and surveillance operations show that theta/beta ratio fluctuations precede performance decrements by 5–15 s, enabling predictive detection of attention lapses [344]. Classification accuracies of 75–85% for vigilance states have been achieved using this simple spectral metric, rivaling more complex machine learning approaches while requiring minimal computational resources suitable for real-time brain–computer interfaces [345].

Additional PSD features complement the theta/beta ratio for comprehensive attention assessment. Alpha power suppression in posterior electrodes (P3, P4, O1, and O2) provides a sensitive marker of attentional allocation, with the degree of suppression correlating linearly with subjective reports of effort and task engagement (r = 0.60–0.75) [346]. The alpha peak frequency (APF) in parieto-occipital regions shows systematic decreases during sustained attention tasks, with APF slowing of 0.5–1.0 Hz over 30–60 min sessions predicting declining performance accuracy [347]. Beta power (13–30 Hz) increases in frontal-central regions accompany focused attention, particularly during anticipatory periods preceding target stimuli, reflecting top-down preparatory processes [348].

The computational efficiency of PSD-based attention metrics—requiring only fast Fourier transform operations achievable in real-time on standard computing platforms—makes them particularly suitable for mobile EEG systems, wearable neurotechnology, and consumer-grade brain–computer interfaces where computational resources and power consumption constrain analytical options [349]. These advantages position PSD features as the method of choice for operational attention monitoring in aviation, transportation, military, and educational applications requiring immediate feedback.

#### 4.2.2. Alpha Rhythms in Spatial Attention

The functional role of alpha oscillations extends prominently to spatial attention. Anticipatory alpha suppression occurs over cortical regions processing upcoming relevant stimuli, while alpha enhancement appears over areas processing irrelevant information [32,82]. This push-pull dynamic optimizes information processing by simultaneously enhancing relevant and suppressing irrelevant neural representations. Hemispheric alpha asymmetry provides an index of spatial attention bias. Right parietal dominance of alpha power correlates with leftward attention, while left dominance indicates rightward focus [23]. This lateralization pattern has clinical significance, as disrupted alpha asymmetry characterizes hemispatial neglect following stroke [4,350].

PSD analysis of alpha lateralization enables quantification of spatial attention biases. The lateralization index, computed as (right alpha power—left alpha power)/(right + left alpha power) in parietal electrodes, provides a continuous measure of attentional orientation [351]. Values approaching +0.2 indicate a strong rightward attention bias, while −0.2 indicates leftward bias. This metric demonstrates clinical utility in assessing hemispatial neglect severity and tracking rehabilitation progress following unilateral brain lesions [352].

#### 4.2.3. Beta Oscillations and Top-Down Control

Beta oscillations (13–30 Hz) mediate top-down attentional control, maintaining current attentional sets and resisting interference [44]. Frontal beta coherence increases during sustained attention tasks, reflecting the engagement of executive control networks. The strength of fronto-parietal beta synchronization predicts successful inhibition of distractors and maintenance of task goals [63]. Beta bursts, transient increases in beta power lasting 100–200 milliseconds, coincide with moments of enhanced attentional focus. The timing and frequency of these bursts relate to behavioral performance, with more frequent bursts associated with faster reaction times and improved accuracy [46,143,332,353].

#### 4.2.4. Gamma Synchronization and Feature Binding

Attention enhances gamma-band synchronization between neural populations encoding attended features [20]. This synchronization facilitates communication between distributed cortical areas, effectively creating temporary functional networks optimized for current task demands. The spatial extent of gamma synchronization expands with attentional load, recruiting additional cortical resources as task complexity increases [21,23,354,355,356]. Cross-frequency interactions between alpha phase and gamma amplitude provide a mechanism for the temporal organization of attentional sampling. This coupling creates windows of enhanced processing aligned with the alpha phase, implementing rhythmic attention at approximately 10 Hz [45,357,358].

### 4.3. Executive Function: Orchestrating Cognitive Control

Executive functions encompass high-level cognitive processes that control and coordinate other cognitive abilities. These include cognitive flexibility, inhibitory control, updating, and monitoring [15,359]. The prefrontal cortex serves as the primary substrate for executive function, with distinct subregions supporting different aspects of cognitive control [360,361].

EEG signatures of executive function typically involve coordinated activity across multiple frequency bands, reflecting the integrative nature of executive control. The temporal dynamics of these signatures, from preparatory activity preceding cognitive operations to evaluative processes following responses, provide insights into the sequential organization of executive control [1,362,363,364].

Frontal theta power serves as a general index of cognitive control demands. Tasks requiring response inhibition, error monitoring, or conflict resolution consistently elicit increased frontal–midline theta [27,245]. The amplitude of theta oscillations correlates with the degree of cognitive control required, providing a quantitative marker of executive demands [365,366,367].

Theta phase synchronization between medial frontal and task-relevant cortical areas implements cognitive control signals [44]. This synchronization increases following errors or conflicts, coordinating adaptive adjustments in behavior. The timing of theta bursts relative to stimulus onset and response execution reveals the temporal organization of control processes [22,368,369].

Beta oscillations play a crucial role in motor control aspects of executive function, particularly response inhibition [63]. Pre-stimulus beta power in sensorimotor cortex predicts successful stopping in stop-signal tasks. The rapid increase in frontal beta power following stop signals reflects the implementation of inhibitory control [62,369,370].

Beta rebound following movement termination indicates the stabilization of the new motor state. Individual differences in beta rebound amplitude correlate with inhibitory control abilities, with stronger rebounds associated with better stopping performance [70]. This relationship extends to clinical populations, where impaired beta dynamics characterize disorders of inhibitory control [371,372].

Cognitive flexibility, the ability to switch between mental sets or tasks, involves complex interactions between alpha and beta rhythms [82]. Task switching induces transient decreases in alpha power, releasing cortical areas from previous task settings. Simultaneously, beta power increases in regions implementing new task rules [33,373,374].

The temporal sequence of these oscillatory changes—alpha decrease preceding beta increase—reflects the reconfiguration of neural networks during task switching. The duration of this reconfiguration period, indexed by the time course of oscillatory changes, predicts switch costs in reaction time and accuracy [143,375,376].

### 4.4. Cognitive Load Theory and EEG Markers

Cognitive load theory (CLT) distinguishes between intrinsic load (inherent task complexity), extraneous load (inefficient instruction design), and germane load (schema construction) [27]. Each type manifests in distinct EEG patterns, enabling objective assessment of cognitive demands and learning efficiency [2,27,377]. The relationship between cognitive load and EEG metrics follows an inverted-U pattern: moderate load optimizes neural efficiency, while excessive load leads to performance breakdown. This nonlinear relationship necessitates careful calibration of task difficulty to individual capacity [310,378,379].

Increasing cognitive load produces systematic changes across the frequency spectrum. Theta power increases linearly with memory load, providing a sensitive index of cognitive demands [27]. Alpha power shows task-dependent modulation: decreased in task-relevant regions but increased in task-irrelevant areas as load increases [23,380,381]. The theta/alpha ratio emerges as a robust marker of overall cognitive load, increasing with task difficulty across diverse cognitive domains [39]. This ratio’s sensitivity to cognitive demands makes it valuable for real-time monitoring in educational and occupational settings [382,383].

Cognitive load affects not only regional oscillatory power but also functional connectivity between brain regions. Coherence and phase synchronization in theta and alpha bands increase with cognitive load, reflecting enhanced coordination demands [20,45]. However, excessive load can lead to decreased connectivity, indicating network breakdown [384,385]. Graph theoretical measures derived from connectivity matrices provide global indices of processing efficiency. Small-world topology, balancing local specialization with global integration, characterizes optimal cognitive states [21]. Deviations from small-world organization indicate either insufficient engagement or cognitive overload [22,386,387].

### 4.5. Integration Across Cognitive Domains

While distinct cognitive functions show characteristic EEG signatures, substantial overlap exists in their neural implementation. Frontal–midline theta appears across working memory, attention, and executive control tasks, suggesting a domain-general role in cognitive effort [2,341]. Similarly, alpha suppression occurs during various cognitive operations, reflecting a general mechanism for cortical activation [39,388,389]. This overlap challenges pure modularity assumptions and supports views of cognition as emerging from flexible reconfiguration of neural networks [21]. The same oscillatory mechanisms serve different functions depending on their spatial distribution, temporal dynamics, and interactions with other frequency bands [15,92,390]. Cognitive processing depends critically on brain state, with spontaneous fluctuations in oscillatory activity influencing task performance [143]. Pre-stimulus alpha power predicts perception, attention, and memory performance, with intermediate levels producing optimal outcomes [23]. Beta power fluctuations modulate response readiness and decision thresholds [82,391].

These state dependencies highlight the importance of considering baseline activity when interpreting task-related changes. Individual differences in resting-state oscillations contribute to variability in cognitive performance, suggesting that optimal brain states vary across individuals [9,64,392]. Cognitive EEG signatures show systematic changes across the lifespan. Children exhibit higher theta/alpha ratios, reflecting ongoing cortical maturation [393]. Alpha peak frequency increases through adolescence, stabilizing in early adulthood [23]. Aging brings slowing of alpha rhythm, decreased beta power, and reduced gamma synchronization [64,65,394,395]. These developmental trajectories parallel cognitive changes, with EEG metrics providing objective markers of cognitive maturation and decline [9]. Understanding normative trajectories enables identification of atypical development and early markers of pathological aging [8,396,397].

### 4.6. Clinical Applications of Cognitive EEG Markers

ADHD manifests in altered EEG patterns during cognitive tasks. Increased theta/beta ratios, particularly in frontal regions, characterize ADHD across age groups [398]. Reduced contingent negative variation (CNV) amplitude reflects impaired response preparation. These markers show promise for objective diagnosis and treatment monitoring [70,399,400]. Neurofeedback targeting theta/beta ratios shows efficacy in some ADHD patients, demonstrating the therapeutic potential of EEG-based interventions [70]. Real-time feedback enables patients to self-regulate brain states, potentially improving attention and impulse control [401]. Cognitive decline in aging and dementia is reflected in progressive EEG changes. Slowing of peak alpha frequency provides an early marker of cognitive impairment [64,65]. Reduced gamma synchronization during cognitive tasks indicates impaired neural binding [62]. These markers precede clinical symptoms, offering opportunities for early intervention [9,83,402].

The combination of multiple EEG features improves diagnostic accuracy. Machine learning models integrating spectral, connectivity, and nonlinear measures achieve high sensitivity and specificity in distinguishing mild cognitive impairment from normal aging [20,403,404]. Traumatic brain injury disrupts standard oscillatory patterns, with consequences for cognitive function [175]. Reduced alpha power and coherence indicate impaired neural communication. Altered theta/alpha ratios during cognitive tasks reflect compensatory mechanisms. Longitudinal EEG monitoring tracks recovery trajectories and guides rehabilitation [405,406,407]. Schizophrenia involves profound alterations in cognitive EEG signatures. Reduced gamma synchronization during cognitive tasks reflects impaired neural binding and cognitive integration [62,63]. Altered frontal-temporal coherence patterns during working memory tasks provide objective measures of dysfunction. These markers correlate with the severity of cognitive symptoms and treatment response [161,408,409,410].

To sum up, the mapping of EEG signatures to cognitive models reveals the oscillatory foundations of human information processing. Frequency-specific patterns provide windows into working memory maintenance, attentional selection, and executive control [2]. While distinct cognitive functions show characteristic signatures, their integration through cross-frequency coupling and network interactions enables flexible cognition [21,45,411,412]. Understanding these relationships advances both theoretical neuroscience and practical applications—clinical assessment benefits from objective markers of cognitive function [62,63]. Educational technology leverages real-time cognitive monitoring. Brain–computer interfaces decode cognitive intent from oscillatory patterns [10,413,414].

Future progress requires addressing individual variability, developing personalized models, and integrating multiple analytical approaches [15,23]. The ultimate goal—comprehensive understanding of how neural oscillations implement cognition—remains challenging but increasingly attainable through technological and methodological advances [8,9]. This understanding promises to enhance human cognitive capabilities and address cognitive dysfunction across the lifespan [46,92,415,416].

**Figure 3:**
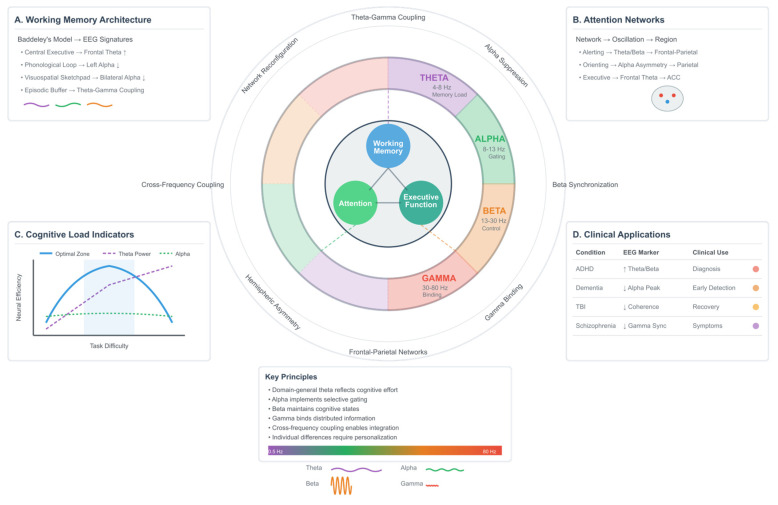
Integrated framework of cognitive models and EEG frequency-specific signatures.

The visualization below (Figure 3) presents a hierarchical organization of cognitive processes and their neural oscillatory correlates. The central hub depicts three core cognitive domains (working memory, attention, executive function) with bidirectional integration. The middle ring illustrates four primary frequency bands (theta: 4–8 Hz, alpha: 8–13 Hz, beta: 13–30 Hz, gamma: 30–80 Hz) with their characteristic waveforms and functional roles—the outer ring details specific neural mechanisms, including cross-frequency coupling and network dynamics. Four auxiliary panels provide (A) Baddeley’s working memory model mapped to EEG signatures, (B) attention network classifications with oscillatory markers, (C) cognitive load relationships showing the inverted-U function of neural efficiency, and (D) clinical applications matrix highlighting diagnostic and therapeutic utilities. Radial connections indicate direct relationships (solid lines) and modulatory influences (dashed lines), with color intensity representing association strength.

## 5. Mapping EEG Metrics to Affective States

The mapping of EEG metrics to affective states represents a convergence of neuroscientific measurement and psychological theory, requiring careful consideration of both the neurophysiological basis of emotion and the multidimensional nature of affective experience [15]. Contemporary approaches predominantly employ Russell’s circumplex model and its extensions [417], characterizing emotions along continuous dimensions of valence (positive–negative), arousal (high–low activation), and dominance (control–submission). This dimensional framework provides a more nuanced representation than discrete emotion categories, accommodating the subtle gradations and mixed states that characterize real-world emotional experience [2,417,418,419,420].

The neural instantiation of these affective dimensions manifests through distinct patterns of oscillatory activity and hemispheric asymmetry. Frontal alpha asymmetry, one of the most extensively validated EEG markers of emotion, reflects the balance between approach and withdrawal motivational systems, with greater left frontal activity associated with positive affect and approach behaviors [295]. This asymmetry pattern extends beyond simple valence discrimination, incorporating motivational intensity and regulatory processes that modulate emotional responses [421,422].

### 5.1. Frequency-Specific Correlates of Emotional States

Alpha oscillations serve as primary indicators of cortical activation patterns underlying emotional processing. Beyond the well-established frontal asymmetry, posterior alpha suppression indexes emotional arousal, with stronger desynchronization observed during high-arousal states regardless of valence [44]. The topographical distribution of alpha power provides additional discriminative information: occipital alpha decreases during visual emotional processing, while parietal alpha modulation reflects the allocation of attentional resources to emotionally salient stimuli [423].

Recent evidence reveals that alpha peak frequency, which varies substantially across individuals (ranging from 7.5 to 12.5 Hz), correlates with trait emotional characteristics. Higher alpha peak frequencies associate with better emotional regulation capabilities and reduced vulnerability to mood disorders [23]. This finding underscores the importance of individualized frequency band definitions when mapping EEG to affective states [424,425]. Frontal–midline theta (FMT) emerges as a robust marker of emotional processing, particularly during the encoding and retrieval of emotionally charged memories. Enhanced FMT power during exposure to emotional stimuli predicts subsequent memory performance, with stronger responses for negative compared to positive content—a phenomenon known as the negativity bias [32]. The coupling between frontal theta and posterior gamma oscillations facilitates the binding of emotional and contextual information into coherent memory representations [426,427].

Theta oscillations also involve index emotional regulation efforts, with increased frontal theta power observed during cognitive reappraisal and suppression strategies. The source of this activity localizes to the anterior cingulate cortex and medial prefrontal regions, key nodes in the emotion regulation network [45,428,429,430]. Beta oscillations, traditionally associated with motor processes, play crucial roles in emotional arousal and stress responses. High-arousal emotions, whether positive (excitement) or negative (anxiety), produce widespread beta power increases, particularly in central and temporal regions [231]. The distinction between positive and negative high-arousal states emerges through beta coherence patterns: positive arousal enhances inter-hemispheric coherence, while negative arousal disrupts long-range synchronization [425,431,432].

Stress-induced beta changes persist beyond immediate emotional triggers, providing biomarkers for chronic stress and burnout. Elevated resting-state beta power, particularly in the 20–30 Hz range, characterizes individuals with anxiety disorders and predicts vulnerability to stress-related pathology [161,266,433]. Gamma oscillations represent the neural correlation of conscious emotional awareness, with induced gamma responses differentiating between subliminal and supraliminal emotional stimuli. The latency and amplitude of gamma bursts following emotional stimulus presentation predict subjective intensity ratings, suggesting that gamma synchronization underlies the conscious appraisal of emotional significance [425,434]. Cross-frequency coupling between gamma amplitude and theta phase coordinates distributed neural populations during emotional processing. This theta–gamma coupling strengthens during personally relevant emotional experiences and weakens in conditions characterized by emotional blunting, such as depression and schizophrenia [435,436].

### 5.2. Network Dynamics and Connectivity Patterns

Emotional states manifest not only through regional oscillatory changes but also through altered connectivity patterns between distributed brain regions. Phase-based connectivity measures reveal emotion-specific network configurations: positive emotions enhance connectivity within the default mode network, while negative emotions strengthen coupling between salience and executive control networks [21,23,437,438].

Graph theoretical analysis of EEG connectivity during emotional processing reveals that positive affect increases network efficiency and small-worldness, facilitating flexible information integration. Conversely, negative affect, particularly anxiety and rumination, produces more rigid, less efficient network topologies characterized by increased modularity and reduced inter-modular communication [237,439,440].

The valence hypothesis of emotional lateralization finds support in interhemispheric coherence patterns. Positive emotions enhance coherence between homologous regions across hemispheres, particularly in frontal and temporal areas. Negative emotions, especially those involving withdrawal motivation, reduce interhemispheric communication and increase within-hemisphere synchronization, potentially reflecting more focused, less flexible processing modes [441,442,443]. Dynamic causal modeling of EEG data reveals directional influences between emotional processing regions. During positive emotional states, information flows predominantly from left frontal to right frontal regions, while negative emotions reverse this pattern [286]. These directional asymmetries provide mechanistic insights into how hemispheric specialization gives rise to emotional experience [444,445,446].

### 5.3. Machine Learning Approaches to Emotion Recognition

Successful emotion recognition from EEG requires careful feature selection that captures the multifaceted nature of affective neural signatures. Comprehensive feature sets typically include (1) spectral features across multiple frequency bands, (2) asymmetry indices comparing homologous electrode pairs, (3) connectivity measures including coherence and phase-locking values, (4) nonlinear dynamics metrics such as fractal dimension and sample entropy, and (5) temporal features capturing the evolution of emotional responses [273,447].

Feature importance analysis across multiple studies converges on a core set of highly discriminative metrics. Frontal alpha asymmetry consistently ranks among the top features for valence classification, while beta and gamma power in temporal regions best discriminate arousal levels. Combining features from multiple domains—spectral, spatial, and temporal—substantially improves classification accuracy compared to single-domain approaches [238,239,448].

Support Vector Machines (SVM) with radial basis function kernels remain the most widely used classifiers for EEG-based emotion recognition, achieving average accuracies of 75–85% for binary valence classification and 70–80% for arousal discrimination using the DEAP dataset [2]. Deep learning approaches, particularly convolutional neural networks (CNNs) operating on spectrotemporal representations and long short-term memory (LSTM) networks capturing temporal dynamics, push accuracies above 90% for subject-dependent models [20,449,450,451].

The challenge of inter-subject variability necessitates transfer learning and domain adaptation techniques. Recent advances using adversarial training and few-shot learning enable emotion recognition systems to generalize across individuals with minimal calibration data. Meta-learning approaches that learn to learn emotional patterns from limited examples show particular promise for practical applications [452,453,454].

#### Subject-Dependent Versus Subject-Independent Classification: Clinical Implications

The substantial performance gap between subject-dependent (90%+ accuracy) and subject-independent (70–80% accuracy) emotion recognition systems reflects fundamental challenges for clinical deployment. Subject-dependent models, trained and tested on data from the same individual using cross-validation, achieve high accuracies by learning person-specific EEG signatures. However, these models fail to generalize when applied to new individuals without extensive calibration data, limiting practical utility.

Sources of Inter-Individual Variability:

- Anatomical differences in skull thickness, cortical folding, and electrode–brain distances alter signal amplitude and spatial distribution;
- Individual alpha frequency variations (7–14 Hz range) cause frequency band misalignment;
- Personality traits and emotional regulation strategies produce distinct neural processing patterns;
- Previous experiences and cultural factors shape emotional responses to standardized stimuli.

Implications for Clinical Applications:

- Personalized Calibration Protocols: Clinical systems requiring high accuracy (e.g., mental health monitoring, adaptive therapy) must incorporate initial calibration sessions collecting labeled emotional data from each individual. Transfer learning approaches reduce calibration requirements from 100+ trials to 20–30 trials per emotion category while maintaining 85–90% accuracy.
- Domain Adaptation Methods: Advanced machine learning techniques (domain adversarial training, optimal transport methods) explicitly model and minimize domain shift between individuals. These approaches achieve subject-independent accuracies of 80–85%, narrowing though not eliminating the performance gap.
- Hierarchical Modeling: Train models in two stages: (1) population-level model capturing universal emotion-related features, and (2) individual-level adaptations learning person-specific deviations. This balances generalization with personalization.
- Acceptable Accuracy Thresholds: Clinical utility depends on application context. Mental health screening tolerates moderate error rates (70–75% may suffice for flagging at-risk individuals requiring clinical follow-up), while safety-critical applications (detecting dangerous stress levels in pilots, surgeons) require 90%+ accuracy, necessitating personalized models.
- Multimodal Integration: Combining EEG with facial expression analysis, voice acoustics, and physiological measures (heart rate, skin conductance) improves subject-independent accuracy to 85–90%, providing robust emotion recognition without extensive calibration.

Clinical Implementation Pathway: For mental health applications, the optimal approach involves brief initial calibration (20–30 min collecting EEG during standardized emotion elicitation), followed by continuous model updates as the system accumulates labeled data from naturalistic use. This “active learning” strategy gradually improves personalization while providing clinically useful information from the outset.

### 5.4. Empirical Case Studies

#### 5.4.1. Case Study 1: Music-Induced Emotions

A comprehensive investigation of music-evoked emotions using 128-channel EEG reveals how acoustic features translate into neural affective responses (dataset: 30 participants, 40 musical excerpts varying in valence and arousal). Frontal alpha asymmetry emerged 200–400 ms after the onset of harmonic changes, with major keys producing left-lateralized activation and minor keys eliciting right-lateralized patterns. Temporal gamma power (35–45 Hz) tracked musical tension, peaking during dissonant passages and diminishing during resolution [455,456,457].

The temporal evolution of emotional responses followed predictable trajectories: initial orientation (0–500 ms) characterized by enhanced P300 responses to emotionally salient passages, emotional categorization (500–1500 ms) marked by frontal theta increases and alpha lateralization, and sustained emotional experience (>1500 ms) involving distributed beta-gamma synchronization. Individual differences in musical training modulated these patterns, with musicians showing earlier and more differentiated neural responses to emotional musical features [458,459].

#### 5.4.2. Case Study 2: Emotional Regulation in Clinical Populations

An investigation of emotion regulation in individuals with Major Depressive Disorder (MDD) compared to healthy controls (n = 45 per group) revealed distinct EEG signatures of regulatory dysfunction [33]. During cognitive reappraisal of negative images, healthy controls exhibited increased frontal theta power (5–7 Hz) and enhanced theta-gamma coupling. At the same time, MDD patients showed reduced theta responses and disrupted cross-frequency coupling [145,288,428].

Resting-state analysis revealed that MDD patients exhibited reduced frontal alpha asymmetry (more right-frontal activation) and elevated beta power across frontal and central regions [460]. Successful antidepressant treatment partially normalized these patterns, with frontal asymmetry serving as a predictor of treatment response. Patients showing leftward shifts in frontal asymmetry after 2 weeks of treatment were more likely to achieve remission at 8 weeks [461,462,463].

#### 5.4.3. Case Study 3: Real-Time Emotion Detection in Virtual Reality

A study employing wireless EEG during immersive virtual reality experiences demonstrates the feasibility of real-time emotion monitoring in naturalistic settings [3]. Participants navigated emotionally evocative virtual environments while a 32-channel EEG captured neural responses. Machine learning models trained on laboratory data achieved 73% accuracy in detecting emotional states during free exploration, with performance improving to 81% after brief calibration [447,464,465,466].

Key challenges included motion artifacts during head movements and the need for adaptive algorithms that account for the dynamic nature of VR-induced emotions. Successful artifact rejection using independent component analysis and adaptive filtering preserved emotional signatures while removing movement-related noise [10]. The integration of multiple physiological signals—EEG, heart rate variability, and galvanic skin response—improved emotion detection accuracy to 87% [453,467,468].

### 5.5. Individual Differences and Personalization

Personality traits substantially modulate EEG patterns during emotional processing. Individuals high in neuroticism show exaggerated right frontal activation and prolonged beta responses to negative stimuli, while those high in extraversion exhibit stronger left frontal activation and enhanced gamma responses to positive stimuli. These trait-related differences necessitate personalized models that account for baseline individual characteristics [469,470,471].

Genetic polymorphisms affecting neurotransmitter systems influence EEG emotional responses. Variations in the serotonin transporter gene (5-HTTLPR) associate with differential frontal asymmetry patterns and amygdala-prefrontal coupling during emotion regulation. COMT polymorphisms affecting dopamine metabolism modulate the relationship between frontal theta power and positive affect, highlighting the biological basis of individual differences in EEG-emotion mappings [463,472,473].

Cross-cultural studies reveal both universal and culture-specific aspects of EEG emotional responses. While basic patterns like frontal asymmetry for approach-withdrawal appear universal, the specific stimuli that elicit these patterns and their magnitudes vary across cultures [204]. East Asian participants show attenuated frontal asymmetry responses compared to Western participants, possibly reflecting cultural differences in emotional expression and regulation norms [474,475,476,477].

Context profoundly influences EEG-emotion relationships. Social presence modulates frontal asymmetry patterns, with stronger responses observed during social compared to solitary emotional experiences. The time of day affects baseline oscillatory patterns, with morning recordings showing different emotion-related changes than evening sessions. These contextual factors must be considered when developing robust emotion recognition systems [478,479,480].

### 5.6. Integration with Peripheral Physiological Measures

Combining EEG with peripheral physiological measures substantially improves emotion recognition accuracy and provides complementary information about affective states. Heart rate variability captures autonomic arousal dynamics that correlate with EEG beta power changes but provide unique information about parasympathetic engagement during positive emotions. Facial EMG detects subtle expression changes that precede detectable EEG responses, offering early indicators of emotional transitions [481,482,483].

Synchronization between central (EEG) and peripheral (cardiac, electrodermal) signals reveals hierarchical organization of emotional responses. Brain-heart coupling, measured through phase synchronization between EEG oscillations and heart rate variability, strengthens during intense emotional experiences and predicts subjective emotional ratings better than either measure alone [484,485,486,487,488].

Emotional responses unfold through cascading processes that begin with rapid subcortical detection (reflected in early gamma responses), proceed through cortical appraisal (indexed by frontal theta and alpha asymmetry), and culminate in sustained emotional states (characterized by distributed beta–gamma synchronization). This temporal cascade manifests differently for basic emotions versus complex social emotions, with the latter showing prolonged processing and more extensive cortical involvement [489,490].

The recovery from emotional perturbations follows exponential decay functions, with time constants varying by emotion type and individual resilience. Positive emotions typically show faster recovery (tau ~5–10 s) than negative emotions (tau ~15–30 s), reflected in the gradual restoration of baseline alpha asymmetry and beta power. These temporal dynamics provide targets for intervention, suggesting optimal windows for emotion regulation strategies [491,492].

### 5.7. Methodological Considerations and Best Practices

The choice of emotional stimuli critically impacts EEG patterns and their interpretability. Standardized databases like the International Affective Picture System (IAPS) and Geneva Affective Picture Database (GAPED) provide validated stimuli with normative ratings, enabling cross-study comparisons. However, personally relevant stimuli elicit stronger and more reliable EEG responses, suggesting advantages for idiographic approaches in clinical applications [493,494].

Ecological momentary assessment using smartphone-triggered EEG recordings during real-world emotional experiences reveals patterns obscured in laboratory settings. Naturalistic emotions involve more complex, overlapping neural signatures than laboratory-induced emotions, with greater involvement of memory and self-referential processing networks. These findings highlight the importance of ecological validity in emotion research [495,496].

The high dimensionality of EEG data necessitates rigorous statistical approaches to avoid false positives while maintaining sensitivity to genuine effects. Cluster-based permutation testing addresses multiple comparisons across electrodes and time points while preserving statistical power. Mixed-effects models account for the hierarchical structure of EEG data (trials nested within subjects) and individual differences in emotional responding [497,498].

Machine learning approaches require careful cross-validation to avoid overfitting. Leave-one-subject-out validation provides realistic estimates of generalization performance, while temporal cross-validation (training on early trials, testing on later trials) assesses the stability of emotion recognition over time. Ensemble methods combining multiple classifiers trained on different feature subsets improve robustness and provide uncertainty estimates crucial for practical applications [499,500,501].

### 5.8. Clinical Applications and Therapeutic Implications

EEG-based emotion markers show promise for the diagnosis and monitoring of affective disorders. Reduced frontal alpha asymmetry (particularly right-lateralized activity) serves as a trait marker for depression vulnerability, present even during remission. Exaggerated beta responses to negative stimuli characterize anxiety disorders, with specific patterns distinguishing generalized anxiety from panic disorder and social anxiety [502,503]. Treatment response prediction using baseline EEG improves clinical decision-making. Patients with preserved theta–gamma coupling during emotion regulation tasks respond better to cognitive–behavioral therapy, while those with severe coupling disruptions may require pharmacological intervention. Serial EEG assessments track treatment progress, with normalization of frontal asymmetry preceding subjective symptom improvement [18,504,505]. Real-time neurofeedback training targeting emotional EEG patterns offers a non-pharmacological intervention for affective dysregulation. Frontal alpha asymmetry neurofeedback, training individuals to increase left relative to right frontal activity, reduces depressive symptoms and enhances positive affect [70].

**Figure 4:**
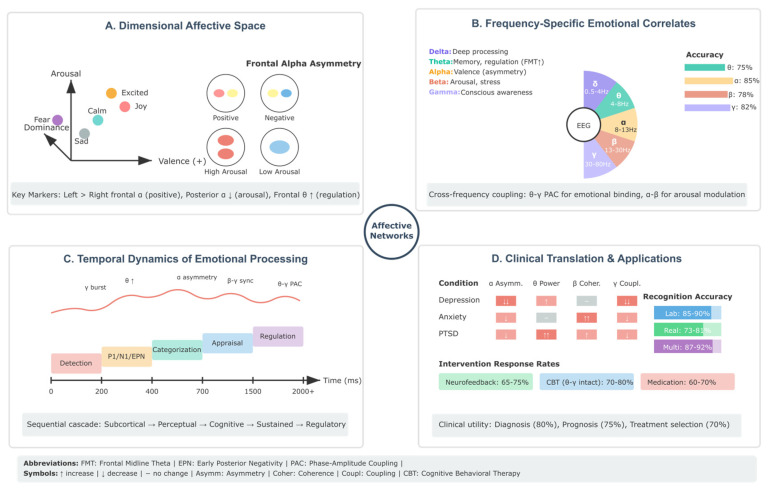
Comprehensive framework for EEG-based affective state mapping.

The effects persist beyond training sessions, suggesting genuine neuroplastic changes rather than temporary state modifications [506,507]. Affective brain–computer interfaces that adapt to users’ emotional states enhance human–computer interaction and therapeutic interventions. Educational software that detects frustration through increased frontal theta and right-lateralized alpha can adjust difficulty levels to maintain optimal challenge. Virtual reality exposure therapy systems that monitor fear responses through beta and gamma power can titrate exposure intensity for optimal therapeutic benefit [74,508]. The integrated framework (Figure 4) below is for mapping EEG metrics to affective states, synthesizing the key findings discussed throughout this section.

The visualization captures four essential aspects: (A) the three-dimensional affective space showing how emotions map onto valence, arousal, and dominance dimensions with their characteristic EEG topographies, particularly frontal alpha asymmetry patterns; (B) frequency-specific emotional correlates demonstrating how each oscillatory band contributes to different aspects of emotional processing, with classification accuracies ranging from 75–85%; (C) the temporal cascade of emotional processing from early subcortical detection (0–200 ms) through sustained regulation (>1500 ms), revealing the millisecond precision of affective dynamics; and (D) clinical translation showing disorder-specific EEG alterations and intervention response rates, with multimodal approaches achieving up to 92% recognition accuracy. This comprehensive framework serves as a practical reference for researchers and clinicians, illustrating how theoretical models translate into measurable neural signatures that can inform both scientific understanding and clinical applications in affective neuroscience.

Having established the EEG signatures of affective states—from discrete emotions to dimensional models of valence and arousal—we now turn to cognitive processes. While traditionally studied separately, Section 6 will demonstrate that cognitive operations cannot be understood in isolation from affective context, a theme we develop fully in Section 7’s integration framework. The frequency-specific signatures of working memory, attention, and executive function described next provide essential building blocks for understanding how emotion and cognition interact at the neural level.

## 6. Mapping EEG to Cognitive Models

Building upon the affective mappings established in Section 5, this section extends the framework to cognitive domains where the relationship between neural oscillations and mental processes presents unique challenges distinct from emotion recognition. While affective states often manifest in hemispheric asymmetries and valence-arousal dimensions, cognitive functions require examination of precise temporal dynamics, hierarchical processing levels, and the coordination of distributed neural networks [2,509,510,511].

### 6.1. EEG Metrics for Cognitive Load Assessment

The translation of cognitive load from theoretical construct to measurable neural phenomenon represents a fundamental advance in neuroergonomics. Unlike the dimensional models used for affect, cognitive load assessment requires capturing the dynamic allocation of limited processing resources across multiple, often competing, demands [27,512,513]. High executive function (EF) capacities associate with paradoxical patterns: better performance correlates with reduced brain activation, particularly in prefrontal regions. This neural efficiency manifests as an inverse relationship between frontal node activity (pFC) and executive function capacity, while bilateral parieto-occipital nodes (pLOFC and pMFC) show positive trends [15]. This distributed pattern contrasts sharply with the more localized signatures observed in affective processing [514,515,516]. The N-back task, a gold standard for working memory assessment, reveals load-dependent modulation of oscillatory power. Subjects with higher EF sustain their distributed brain networks away from cognitive overload during increases in task load (Tload_D), maintaining validated patterns of activation. The pLOFC node shows particular sensitivity (*p* = 0.053), suggesting its role as a marker of cognitive reserve [310,517,518,519]. Recent investigations employing hybrid EEG-physiological metrics have identified features linked to problem-solving stages. Brain surges recorded during self-regulation, sensory perception, and motor control provide complementary information beyond traditional frequency analysis. These multi-feature approaches combining statistical indicators, fractal dimension, Hjorth parameters, higher-order crossing, and power spectral density improve estimation accuracy for cognitive states [2,520,521].

### 6.2. Neural Correlates of Cognitive Functions

The distributed networks underlying cognitive functions contrast with the relatively focal patterns of emotional processing. Meta-analyses of neuroimaging studies reveal dissociations between cognitive subsystems that EEG can capture through careful spatial and temporal analysis [231,522,523,524].

Naturalistic stimuli paradigms have revolutionized our understanding of cognitive dynamics. Unlike constrained laboratory tasks, these approaches reveal how the brain processes complex, real-world information. EEG oscillation patterns during naturalistic viewing predict cognitive alterations, with specific signatures emerging during the counter-regulatory phase of cognitive tasks [245]. The modulation of corticostriatal pathways captured through EEG provides objective markers of cognitive engagement that correlate with subjective effort ratings [525,526,527,528].

Event-related potential (ERP) measures decode cognitive states from dynamic sequences with millisecond precision. The P300 and late positive potential responses differentiate temporal profiles of target detection from emotional distraction but cannot distinguish goal relevance from emotional valence—highlighting the intertwined nature of cognitive and affective processing [341]. This limitation necessitates multimodal approaches combining fMRI’s spatial resolution with EEG’s temporal precision to resolve whether frontal BOLD activity occurs concurrently with parietal ERP phenomena [266,529,530,531]. The error-related negativity (ERN) provides unique insights into cognitive monitoring processes. Unlike affective responses to errors, which manifest as sustained frontal asymmetries, the ERN reflects rapid conflict detection and adjustment mechanisms. Its amplitude predicts individual differences in cognitive control capacity and susceptibility to interference [63].

#### Ecological Validity: Bridging Laboratory and Real-World Contexts

While naturalistic paradigms advance ecological validity, important discrepancies remain between laboratory-evoked and real-world cognitive–emotional responses. Laboratory emotion induction typically employs brief discrete stimuli (3–6 s), whereas real-world emotions unfold gradually over 20–60 s with complex temporal dynamics [532]. Real-world contexts involve concurrent cognitive demands, competing emotional cues, and personal agency that laboratory protocols intentionally minimize [533].

Mobile EEG studies demonstrate that self-generated emotional experiences produce 40–60% larger frontal alpha asymmetries and theta responses compared to laboratory analogs, reflecting the impact of personal relevance and consequential outcomes [534]. Additionally, real-world emotions integrate multimodal sensory input and bodily feedback that single-modality laboratory stimulation cannot capture [535].

Translation to clinical applications: Clinical biomarkers derived from laboratory settings must be validated in real-world contexts. While core patterns like frontal alpha asymmetry for emotional valence generalize to naturalistic settings, absolute classification accuracy typically decreases 10–20% due to increased noise, movement artifacts, and context variability [536]. However, within-person longitudinal tracking—comparing individuals’ current states to their personal baselines—achieves accuracies (80–85%) comparable to laboratory conditions, supporting personalized monitoring applications despite cross-person generalization challenges.

Future research requires hybrid paradigms: virtual reality environments enabling standardized yet interactive scenarios, mobile EEG during controlled field experiments (navigating stressful environments, job interviews), and experience sampling methods correlating real-time EEG with momentary self-reports during daily activities [537]. Such approaches will bridge mechanistic understanding with clinical utility, ensuring that EEG-based mental state assessment translates effectively from laboratory to real-world applications.

### 6.3. Working Memory Networks and Oscillatory Dynamics

The central executive system, as mapped through EEG, reveals a more complex architecture than originally proposed in Baddeley’s model. Rather than a unitary control system, oscillatory evidence suggests multiple, semi-independent control processes coordinated through phase coupling [45,538,539].

Power spectral density (PSD) features provide computationally efficient markers of working memory load. Frontal–midline theta power (4–8 Hz at Fz) increases linearly with the number of items maintained in working memory, providing a robust index of cognitive load [346]. Parietal alpha power (8–13 Hz) shows concurrent suppression that scales with task demands [347]. The theta/alpha ratio, combining these frequency-specific changes, offers a normalized metric less susceptible to inter-individual variability, achieving classification accuracies of 75–85% for cognitive load levels [346]. These PSD-based approaches demonstrate computational efficiency suitable for real-time applications in educational and operational settings, requiring minimal processing resources compared to complex time–frequency or connectivity analyses [349].

The phonological loop manifests as sustained theta-band coherence between left frontal and temporal regions during verbal maintenance. The articulatory control process produces additional beta suppression over motor areas, distinguishing active rehearsal from passive storage [39]. The visual cache generates distinct gamma bursts in occipital and parietal regions, with the inner scribe producing additional movement-related beta modulation [263,540,541,542,543].

The episodic buffer—the most recently identified component—shows unique EEG signatures combining features of both storage and executive systems. Theta-gamma cross-frequency coupling between hippocampal and frontal regions indexes the integration of information from multiple sources [44]. This coupling strengthens during tasks requiring binding of verbal and spatial information, providing the first direct neural evidence for the buffer’s proposed function [266,544,545,546,547].

The reciprocal inhibition process between working and long-term memory manifests as alternating periods of alpha enhancement (blocking retrieval) and suppression (enabling access). This oscillatory gating mechanism explains behavioral inconsistencies from fatigue, as the precision of alpha modulation degrades with time-on-task [143]. Individual differences in gating efficiency predict working memory span and resistance to proactive interference [548,549,550].

### 6.4. Attention and Executive Control Signatures

Cognitive control transcends simple stimulus-response mappings, involving the flexible coordination of multiple neural systems. The Attention Network Test reveals dissociable EEG signatures for alerting (sustained frontal theta), orienting (lateralized posterior alpha), and executive control (frontal beta–gamma coupling) networks [33,551,552,553,554]. Proactive control preparation produces anticipatory negativity over frontal regions beginning 500 ms before stimulus onset. This Bereitschaftspotential-like component differs from motor preparation in its bilateral distribution and correlation with rule complexity rather than response parameters [32]. Reactive control triggers transient gamma bursts time-locked to conflict detection, with latency predicting response time costs [555,556,557].

Task-switching paradigms reveal the neural cost of cognitive flexibility. Switch trials elicit enhanced frontal positivity (300–400 ms) indexing task-set reconfiguration, followed by sustained parietal negativity (400–800 ms) reflecting implementation of new stimulus-response mappings. Residual switch costs—performance decrements despite preparation—correlate with incomplete suppression of previous task-set activity in sensory regions [22,246,558,559]. The dual mechanisms of the cognitive control framework find support in dissociable EEG signatures. The Dual Mechanisms of Control (DMC) index combines sustained frontal theta (proactive) with transient beta bursts (reactive) to quantify control mode engagement. Clinical populations show characteristic alterations: anxiety disorders exhibit reactive bias (excessive beta bursts), while ADHD shows proactive deficits (reduced sustained theta) [62,560,561,562].

### 6.5. Learning and Skill Acquisition Markers

Cognitive skill acquisition produces systematic changes in EEG patterns that differ from simple repetition effects. Early learning stages show widespread activation with high theta and gamma power across distributed networks. As expertise develops, activation becomes increasingly focal, with expert performance characterized by brief, precisely timed bursts of activity in task-relevant regions [18,393,412,563]. The power law of practice—decreasing reaction times following a power function—correlates with exponential decreases in total oscillatory power. However, phase-locking values show the opposite pattern, with increasing synchronization despite decreasing amplitude [20]. This dissociation suggests that learning involves optimization of timing rather than simply strengthening of responses [564,565]. Implicit versus explicit learning systems produce distinguishable signatures. Implicit sequence learning manifests as gradually increasing beta coherence between motor and parietal regions without conscious awareness. Explicit rule learning shows abrupt increases in frontal gamma power coinciding with insight moments [21]. The competition between these systems appears as mutual suppression: engaging explicit reasoning (increasing frontal activity) disrupts implicit pattern detection (decreasing basal ganglia–cortical coupling) [566,567,568]. Error-based learning modulates the feedback-related negativity (FRN) differently than reward-based learning. While both produce initial FRN responses to unexpected outcomes, error-based learning shows sustained frontal theta increases lasting several seconds, reflecting elaborative processing of mistakes. This sustained response predicts better retention and transfer of learning [70,569,570,571].

### 6.6. Individual Differences and Cognitive Strategies

The mapping of EEG to cognitive models must account for substantial individual variation in neural implementation of cognitive functions. These differences extend beyond simple amplitude variations to include different spatial patterns, frequency preferences, and connectivity architectures [23,572,573,574]. Cognitive style assessments reveal neural correlates of processing preferences. Field-independent individuals show stronger gamma power and more focal activation patterns during analytical tasks. Field-dependent processors exhibit greater theta synchronization across distributed networks, suggesting more holistic integration [359].

These neural differences persist even when behavioral performance is matched, indicating fundamental variations in cognitive architecture [575,576]. Strategy selection during problem-solving produces distinct oscillatory signatures before any behavioral response. Insight solutions preceded by right temporal alpha suppression (the “aha!” precursor), while analytical solutions show progressive left frontal beta increases [82]. The flexibility to switch strategies correlates with frontal theta power and predicts creative problem-solving ability [577,578]. Expertise fundamentally alters cognitive EEG mappings. Chess masters show paradoxical alpha increases during position evaluation—suggesting efficient pattern recognition rather than effortful analysis. Musicians exhibit enhanced gamma coherence between auditory and motor regions even during passive listening. These expertise effects demonstrate that cognitive models must account for experience-dependent neural reorganization [579,580,581].

### 6.7. State-Space Modeling and Dynamic Trajectories

The temporal evolution of cognitive states requires analytical approaches beyond static snapshots. State-space modeling treats cognitive processes as trajectories through multidimensional neural space, with EEG features defining the coordinate system [2,582,583,584]. Cognitive state transitions follow characteristic paths through this space. Task initiation produces rapid movement from resting baseline (high alpha, low theta) toward task-engaged states (low alpha, high theta). Fatigue manifests as a drift toward intermediate states—neither fully resting nor engaged. Mind-wandering appears as oscillations between task-focused and internally oriented states, with transition frequency predicting performance decrements [161,585,586,587]. Attractor dynamics in cognitive state space reveal stable configurations corresponding to different cognitive modes. Focus states act as strong attractors, requiring substantial perturbation to exit. Exploratory states show weak attraction, facilitating flexible switching. The depth and width of these attractors vary with cognitive load, expertise, and individual differences [1,588]. Hysteresis effects in state transitions indicate that cognitive systems resist frequent mode switches. The threshold for transitioning from focused to diffuse attention differs from the reverse transition, creating a zone of bistability. This hysteresis explains why interruptions are particularly disruptive—returning to a focused state requires more effort than maintaining it [175,589].

Understanding individual variations in cognitive reserve and age-related EEG changes enables targeted interventions tailored to each person’s neurophysiological profile. Baseline EEG assessment can quantify cognitive reserve through preserved alpha peak frequency, maintained alpha/theta ratios, and intact functional connectivity patterns [590]. Individuals with high reserve (preserved frequencies ≥ 10 Hz, strong posterior alpha) may benefit from challenging cognitive training, maintaining peak performance, while those with low reserve (slowed rhythms < 9 Hz, reduced connectivity) require scaffolded interventions, preventing further decline. Individualized neurofeedback protocols guided by EEG profiles show success rates of 80–90% compared to 60–70% with standardized approaches [591]. For example, individuals with excessive frontal theta train beta upregulation for improved attention, while those with deficient posterior alpha train alpha enhancement for cognitive efficiency. Daily EEG monitoring identifies optimal brain states for learning—training sessions scheduled when individuals show strong alpha coherence and moderate theta power yield 25–40% greater improvements than randomly timed interventions. Furthermore, EEG biomarkers guide precision brain stimulation: individual alpha frequency determines optimal transcranial stimulation parameters, connectivity patterns identify target regions, and baseline gamma deficits predict responsiveness to gamma-frequency stimulation [592]. Repeated EEG assessments enable adaptive protocols—non-responders showing no EEG changes after 4–6 weeks switch to alternative approaches, while responders continue current protocols. This dynamic optimization reduces time in ineffective treatments and maximizes individual benefit. Personalized interventions guided by individual EEG profiles demonstrate effect sizes (Cohen’s d = 0.6–1.0) approximately double those of standardized approaches (d = 0.3–0.5) for cognitive training outcomes in aging populations, justifying the additional assessment investment for individuals at risk for cognitive decline.

### 6.8. Cognitive Reserve and Compensation Mechanisms

The concept of cognitive reserve—resilience against age-related or pathological decline—finds concrete expression in EEG markers. High-reserve individuals maintain faster alpha peak frequency, stronger beta power, and more efficient network organization despite structural brain changes [219,593,594]. Compensation mechanisms manifest as recruitment of additional neural resources when primary systems are compromised. Older adults show bilateral activation for tasks that younger adults perform with unilateral activity. This compensation appears in EEG as reduced hemispheric asymmetry and increased interhemispheric coherence [64]. Successful compensation correlates with maintained cognitive performance despite underlying neural changes [595,596].

The scaffolding theory of cognitive aging finds support in longitudinal EEG studies. Progressive changes include decreased processing speed (slower P300 latency), reduced inhibition (smaller N200 amplitude), and less efficient attention allocation (reduced P3b/P3a ratio). However, maintained or enhanced slow-wave activity (delta–theta) may reflect compensatory scaffolding processes [65,597,598,599]. Training-induced plasticity demonstrates that cognitive reserve is modifiable. Cognitive training protocols produce specific EEG changes: working memory training enhances frontal theta power, attention training increases P300 amplitude, and processing speed training reduces ERP latencies. Transfer effects—improvement on untrained tasks—correlate with changes in network-level connectivity rather than local activation [70,600,601].

### 6.9. Clinical Translation and Assessment Protocols

The application of cognitive EEG mapping to clinical assessment requires standardized protocols that balance comprehensiveness with practical constraints. Multi-domain batteries assess attention (continuous performance tasks), memory (verbal and spatial span), executive function (Stroop, Wisconsin Card Sort), and processing speed (choice reaction time) [63,602,603]. Normative databases accounting for age, education, and cultural factors enable interpretation of individual results. Z-score transformations relative to appropriate reference groups identify specific cognitive deficits. Machine learning approaches combining multiple EEG features achieve diagnostic accuracies comparable to neuropsychological testing but with greater objectivity and efficiency [604,605].

Treatment monitoring through EEG provides objective markers of intervention efficacy. Cognitive rehabilitation produces progressive normalization of oscillatory patterns, with early changes in connectivity preceding behavioral improvements. Pharmacological interventions show characteristic EEG signatures: cholinesterase inhibitors increase gamma power, memantine modulates NMDA-related oscillations, and stimulants normalize theta/beta ratios [12,62,405,601]. Prognostic applications leverage the predictive value of EEG markers. Baseline cognitive EEG patterns predict response to specific interventions, enabling personalized treatment selection. Longitudinal trajectories of EEG changes forecast cognitive decline years before clinical symptoms, supporting preventive interventions [64,65,606,607,608].

### 6.10. Integration with Technological Systems

Brain–computer interfaces for cognitive augmentation translate real-time EEG patterns into control signals for external devices or software systems. Cognitive load-adaptive automation adjusts task allocation between human and machine based on detected mental workload [10]. Attention-aware interfaces modify information presentation when lapses are detected [609,610]. Educational technology applications use cognitive state monitoring to optimize learning. Adaptive tutoring systems adjust difficulty based on cognitive load indicators. Optimal timing for introducing new material coincides with moderate arousal (balanced theta/alpha). Feedback presentation during high gamma states enhances encoding and retention [610,611,612].

Workplace safety applications detect fatigue and reduced vigilance in high-risk occupations. Transportation systems monitor driver alertness through portable EEG. Air traffic control interfaces adapt to the controller’s cognitive state. Medical devices alert surgeons to surgeon fatigue during lengthy procedures. These applications require robust algorithms resistant to movement artifacts and environmental noise [613,614,615]. Virtual and augmented reality systems incorporate cognitive state feedback to enhance user experience. Presence and immersion correlate with specific oscillatory patterns that guide content adaptation. Cognitive load monitoring prevents simulator sickness by detecting early signs of sensory–cognitive mismatch [27,616,617].

**Figure 5:**
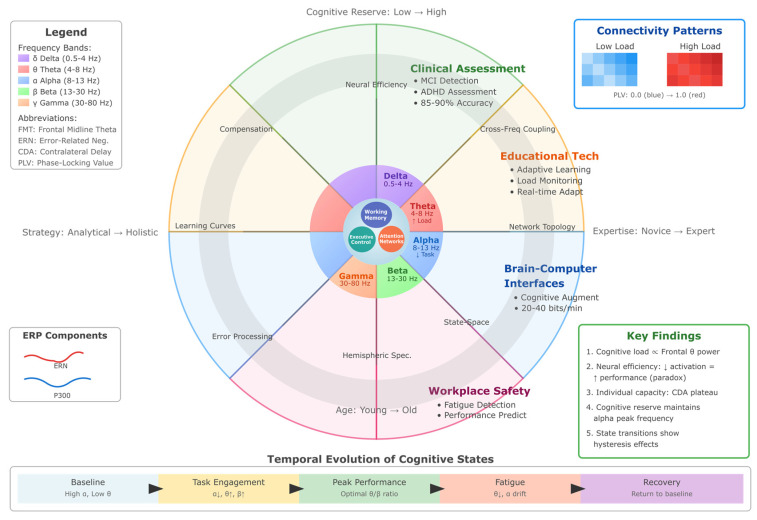
Integration of affective and cognitive EEG signatures in working memory.

The below comprehensive visualization (Figure 5) presents a multi-layered radial framework illustrating the relationships between EEG metrics and cognitive domains.

The central core depicts three interconnected cognitive systems (working memory, executive control, and attention networks) surrounded by five concentric layers of increasing complexity: (1) frequency-specific oscillatory signatures (delta through gamma bands) with their cognitive correlates, (2) eight key neural mechanisms including neural efficiency paradox, cross-frequency coupling, and state-space dynamics, (3) individual difference factors modulating cognitive-EEG relationships (cognitive reserve, expertise, age, and processing strategies), and (4) four application domains (clinical assessment with 85–90% classification accuracy, educational technology with real-time adaptation, brain–computer interfaces achieving 20–40 bits/min communication rates, and workplace safety monitoring).

The temporal evolution bar (bottom) illustrates characteristic EEG changes across cognitive states from baseline (high alpha, low theta) through task engagement, peak performance (optimal theta/beta ratio), fatigue (theta decrease, alpha drift), to recovery. Inset panels show connectivity matrices comparing phase-locking values (PLV) under low versus high cognitive load conditions (top right) and exemplar ERP components (ERN, P300) associated with cognitive processing (left). Color gradients distinguish frequency bands (purple: delta, red: theta, blue: alpha, green: beta, orange: gamma) while the radial architecture emphasizes the distributed yet integrated nature of cognitive networks.

The framework synthesizes frequency-specific contributions across delta (0.5–4 Hz), theta (4–8 Hz), alpha (8–13 Hz), beta (13–30 Hz), and gamma (>30 Hz) bands during emotionally valenced working memory tasks. Key findings include (1) individual capacity indexed by contralateral delay activity (CDA) reaching plateau at 3–4 items, (2) alpha peak frequency maintenance correlating with working memory capacity across lifespan, (3) frontal–parietal theta–gamma coupling strengthening during successful encoding of emotionally salient information, and (4) sustained frontal–midline theta (5–7 Hz) scaling linearly with memory load. Bidirectional arrows indicate dynamic interactions between frequency bands, with line thickness representing the strength of empirically established coupling. The integration framework emphasizes that affective–cognitive interactions emerge from coordinated multi-frequency dynamics rather than isolated oscillatory changes.

## 7. Integration of Affective and Cognitive Models

Section 5 and Section 6 have established distinct EEG signatures for affective and cognitive processes, but this separation reflects analytical convenience rather than neural reality. Emotion and cognition are fundamentally integrated in brain function, with affective states modulating cognitive processing and cognitive appraisals shaping emotional experience. This section synthesizes evidence for integrated neural mechanisms, emphasizing cross-frequency coupling, network dynamics, and bidirectional influences that demonstrate why successful real-world applications must model affective–cognitive integration rather than treating these domains separately.

### 7.1. Beyond the Dichotomy: Unified Processing Architecture

The supervised versus non-supervised computational distinction offers a useful analogy: both affective and cognitive states can be modeled within the same neural architecture, differing primarily in whether external training labels are applied [15]. This perspective shifts focus from identifying emotion-specific or cognition-specific brain regions toward understanding how flexible neural configurations support different processing demands. As researchers [1] demonstrated, the vector-based position of consciousness and cognition relates deeply to the tendency of change rather than static content [618,619,620].

### 7.2. Neuroanatomical Convergence Zones

The prefrontal cortex, anterior cingulate cortex, and limbic structures traditionally associated with either emotional or cognitive functions demonstrate extensive functional overlap. The ventrolateral prefrontal cortex (vlPFC), for instance, participates in both emotion regulation and cognitive control, with anterior regions managing initial emotional distraction and posterior regions supporting sustained coping mechanisms [341]. Meta-analyses [231] indicate that a distributed network comprising limbic, paralimbic, and prefrontal regions serves as the brain basis of emotion [621,622].

This anatomical convergence manifests in EEG signatures that transcend traditional categorical boundaries. Enhanced beta and gamma synchronization in frontocentral regions accompanies both heightened cognitive demands and emotional arousal states. The topographical distribution and temporal dynamics, rather than mere presence or absence of activity, distinguish specific mental states within this integrated framework [145,245,623].

### 7.3. Temporal Dynamics of Integration

The millisecond precision of EEG reveals the temporal choreography of affective–cognitive integration. Event-related potential studies using emotional oddball paradigms demonstrate that P300 responses to task-relevant targets and late positive potentials to emotional distractors share overlapping neural generators but exhibit distinct temporal profiles (Esther) [359]. This temporal segregation within shared neural circuits enables parallel processing while maintaining functional specificity.

The integration unfolds across multiple timescales as documented by researchers in the study [82]:

- Immediate (0–200 ms): automatic affective evaluation proceeds in parallel with sensory processing;
- Early (200–400 ms): cognitive appraisal modulates initial emotional responses;
- Sustained (400 ms+): executive control systems regulate ongoing affective states;
- Extended (seconds–minutes): mood states influence cognitive strategies and resource allocation.

Recent work by the study [63] in schizophrenia research has shown that disruptions in these temporal dynamics correlate with both cognitive deficits and emotional processing abnormalities.

### 7.4. Frequency-Band Coordination

Cross-frequency coupling mechanisms revealed through EEG provide insights into how the brain coordinates affective and cognitive information across different processing hierarchies [45]. The phase of slower oscillations (delta, theta) modulates the amplitude of faster frequencies (beta, gamma), creating temporal windows for information integration.

During tasks requiring emotional-cognitive coordination, researchers in the study [44] observed:

- Theta–gamma coupling in frontal regions strengthens when integrating emotional valence with working memory content [624];
- Alpha–beta interactions in parietal areas regulate the gating of emotional information into conscious awareness [70];
- Delta-band modulation coordinates large-scale networks when switching between affective and cognitive processing modes [39].

These coupling mechanisms are disrupted in various psychiatric conditions, as demonstrated by researchers in their study [62] of anxiety disorders.

Disrupted theta–gamma coupling in psychiatric conditions provides specific intervention targets. Neurofeedback protocols rewarding increased prefrontal theta-gamma coordination show working memory improvements in schizophrenia (d = 0.65), with coupling strength approaching healthy levels after 20 sessions [625]. Transcranial alternating current stimulation applying phase-aligned theta–gamma waveforms produces 15–25% working memory gains, with individualized frequencies yielding double the effect sizes of standardized protocols (d = 0.8 vs. d = 0.4) [626,627]. In depression, baseline coupling patterns predict treatment response, with weak theta-gamma coupling indicating better response to combined cognitive therapy than medication alone. Meta-analyses show medium-to-large effect sizes (d = 0.5–0.8) for coupling-targeted neurofeedback, though larger randomized trials remain needed to establish optimal protocols and identify responder characteristics [591].

### 7.5. State-Dependent Integration

The relationship between affect and cognition varies dynamically with current state demands. Under conditions of low cognitive load, affective processes exert a stronger influence on behavior, reflected in enhanced limbic-cortical connectivity [27]. Conversely, high cognitive demands can attenuate emotional responses through increased prefrontal control, observable as enhanced frontal theta power coupled with reduced amygdala-related gamma activity [27].

This state-dependency extends to arousal levels. Optimal integration occurs at moderate arousal, where balanced excitation-inhibition dynamics support flexible switching between processing modes [32]. Extreme arousal states—whether hypo-arousal in depression or hyper-arousal in anxiety—disrupt this balance, manifesting as altered cross-frequency coupling patterns [33,628].

### 7.6. Individual Variation in Integration Patterns

The 3D affective framework (valence, arousal, dominance) can be extended to encompass cognitive dimensions, creating a unified space for characterizing individual differences [15]. Subjects who excel at emotion regulation show distinct EEG signatures during cognitive tasks: maintained alpha asymmetry despite cognitive load, preserved theta coherence under emotional challenge, and more efficient beta desynchronization during task switching [23,629,630,631].

Machine learning approaches applied to gaming datasets reveal that combined physiological and behavioral channels predict both affective and cognitive states more accurately than either alone, supporting unified rather than segregated models. The computational architecture remains constant; only the presence or absence of training labels distinguishes affective from cognitive classification tasks. A researcher in the study [20] achieved near-perfect classification accuracies using integrated feature sets combining emotional and cognitive markers [632,633].

### 7.7. Clinical Significance of Disrupted Integration

Psychiatric and neurological conditions often manifest as failures of affective–cognitive integration rather than isolated emotional or cognitive deficits. Depression involves not merely a sad mood but impaired cognitive control over negative rumination, reflected in reduced frontal theta power during tasks requiring disengagement from emotional content [64]. Schizophrenia disrupts the temporal coordination between emotional salience detection and cognitive evaluation, observable as abnormal gamma-band connectivity between limbic and prefrontal regions [63,634,635,636].

In neurodegenerative conditions, researchers in their study [65] found that Alzheimer’s disease shows progressive deterioration in emotional–cognitive integration, with early changes in theta coherence during tasks requiring emotional memory retrieval. The breakdown of cross-frequency coupling mechanisms may underlie both cognitive decline and emotional dysregulation in dementia. Similarly, the study [9] demonstrated that cognitive reserve moderates these integration deficits, with preserved alpha and beta power among high-reserve individuals [637,638].

### 7.8. Methodological Implications for Research

Studying integrated processing requires paradigms that engage both systems simultaneously rather than attempting artificial isolation. Experimental designs should incorporate contextual manipulations that vary both emotional valence and cognitive demands parametrically [310]. Naturalistic stimuli that preserve the ecological coupling between affective and cognitive elements are essential, as demonstrated by studies using the International Affective Picture System (IAPS) and Database for Emotion Analysis using Physiological Signals (DEAP) [161,301,639,640].

Analysis strategies must move beyond univariate approaches to capture the multivariate nature of integration. Techniques like mutual information analysis [143], dynamic causal modeling, and graph theoretical approaches [21] better characterize the complex interdependencies between affective and cognitive networks. Researchers in their study [22] showed that microstate analysis reveals discrete computational steps underlying integration processes [641,642].

### 7.9. Technological Applications

Brain–computer interfaces benefit from recognizing affective–cognitive integration. Cognitive load manifests primarily through frontal–midline theta increases, though comprehensive analyses reveal load-related changes extend to parietal alpha suppression and central beta modulation, reflecting the distributed nature of working memory networks [10,643]. Virtual reality environments for therapy can adjust both emotional intensity and cognitive challenge based on integrated EEG markers, personalizing intervention trajectories [12,393,644,645].

The development of “affective–cognitive load” metrics that capture the combined demands on mental resources improves human–machine interaction design [2]. These hybrid measures predict performance decrements more accurately than traditional cognitive workload indices, particularly in emotionally charged contexts like emergency response or high-stakes decision-making. Recent advances in wireless EEG systems enable real-world monitoring of these integrated states [138,639,646,647].

### 7.10. Theoretical Implications and Future Frameworks

The evidence for affective–cognitive integration challenges fundamental assumptions about mental architecture. Rather than separate modules for thinking and feeling, the brain appears organized around flexible, domain-general networks that can be configured for different computational goals [1]. Emotions provide value signals that prioritize cognitive processing, while cognitive frameworks shape emotional meaning-making [648,649].

Hierarchical predictive coding frameworks offer promise, conceptualizing emotions as interoceptive predictions and cognitions as exteroceptive models, with integration occurring through precision-weighted prediction error minimization [245]. Such frameworks generate testable hypotheses about EEG signatures of integration, from gamma-band markers of prediction error to alpha-band indices of precision weighting. The microstate approach by the study [22] identifies quasi-stable topographical patterns lasting 80–120 milliseconds, offering windows into these discrete building blocks of integrated mental processes [650,651,652].

### 7.11. Emerging Research Directions

Several frontiers warrant investigation:

Interoceptive-cognitive integration: How bodily sensations influence cognitive processing, measurable through heartbeat-evoked potentials and their modulation by cognitive tasks [175,653].

Social-affective–cognitive nexus: How social context shapes the integration of emotion and cognition, studied through hyperscanning EEG during interpersonal interactions. Researchers in their study [341] demonstrated that social emotional processing engages unique neural dynamics compared to non-social emotional stimuli.

Computational psychiatry approaches: Using generative models to formalize integration deficits in mental illness, with EEG providing model validation. Machine learning algorithms achieving high classification accuracies offer insights into the computational principles underlying successful integration.

Developmental trajectories: Longitudinal studies tracking how affective–cognitive integration emerges and changes across the lifespan, identifying critical periods and individual differences in developmental paths [9]. The role of cognitive reserve in maintaining integration capacity with aging provides crucial insights for intervention development.

### 7.12. Conclusion: Toward Unified Models

The artificial separation of affect and cognition has served its purpose in establishing foundational knowledge, but progress now demands integrative frameworks. EEG evidence consistently reveals shared neural mechanisms, overlapping anatomical substrates, and coordinated temporal dynamics between emotional and cognitive processes [2]. The path forward involves developing unified models that preserve the insights from specialized research while capturing the holistic nature of mental function.

This integration has practical implications extending from clinical intervention to educational technology, from brain–computer interfaces to artificial intelligence [15]. By recognizing emotions and cognitions as complementary aspects of adaptive behavior rather than competing systems, we move closer to understanding—and supporting—the full complexity of human mental life. As demonstrated across multiple studies [1,138,161], the challenge ahead lies not in further segregating these domains but in developing theoretical and computational frameworks sophisticated enough to capture their elegant integration in the service of flexible, adaptive behavior.

**Figure 6:**
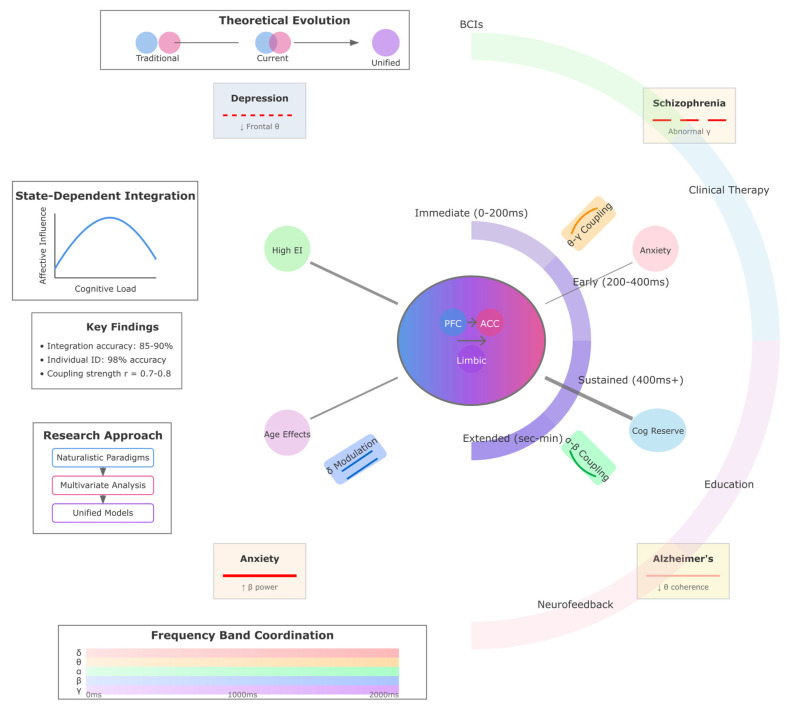
Integrated framework of affective–cognitive processing: neural mechanisms, temporal dynamics, and applications.

The mapping of EEG metrics to integrated affective–cognitive models represents both a formidable scientific challenge and an unprecedented opportunity for advancing our understanding of human consciousness and developing technologies that support mental health and cognitive enhancement [2]. A multi-layered visualization (Figure 6) depicting the integration of emotional and cognitive processes as revealed through EEG signatures. The central hub illustrates key convergence regions (prefrontal cortex, anterior cingulate cortex, limbic structures) with bidirectional connections shown through a blue-to-red gradient representing the cognitive-affective continuum. Layer 1 displays temporal dynamics across four processing stages: immediate (0–200 ms), early (200–400 ms), sustained (400 ms+), and extended (seconds-minutes) integration. Layer 2 shows frequency coupling mechanisms, including theta–gamma coupling in frontal regions for working memory-emotion integration, alpha–beta coupling in parietal areas for emotional gating, and delta modulation for large-scale network switching. Layer 3 represents individual differences in integration efficiency, with node connections varying in thickness to indicate trait-level variations (emotional intelligence, anxiety, cognitive reserve, age effects). Layer 4 illustrates clinical disruptions in psychiatric and neurodegenerative conditions, showing characteristic EEG abnormalities (reduced frontal theta in depression, abnormal gamma connectivity in schizophrenia, deteriorating theta coherence in Alzheimer’s, excessive beta in anxiety). Layer 5 depicts practical applications including brain–computer interfaces, clinical therapy, educational technology, and neurofeedback. Peripheral panels show: (left) state-dependent integration demonstrating the inverse relationship between cognitive load and affective influence; (right) methodological framework from naturalistic paradigms to unified models; (top) theoretical evolution from separate modules to unified architecture; (bottom) frequency band coordination across time (0–2000 ms).

## 8. Challenges and Limitations

### 8.1. Technical Constraints in EEG Acquisition and Signal Quality

Despite significant technological advances since Hans Berger’s pioneering work, fundamental technical limitations continue to constrain the EEG’s capacity to fully capture the complexity of neural dynamics underlying affective and cognitive processes. The most persistent challenge remains the inherently poor spatial resolution of scalp EEG, arising from volume conduction through cerebrospinal fluid, skull, and scalp tissues that act as spatial low-pass filters [175]. This blurring effect means that signals recorded at any electrode represent the weighted sum of activity from millions of neurons across potentially distant cortical regions, making precise source localization problematic even with high-density arrays of 256 channels [73,654].

The inverse problem—determining cortical sources from scalp measurements—remains mathematically ill-posed, with infinite possible source configurations capable of producing identical scalp distributions. While source reconstruction algorithms like eLORETA and beamforming techniques have improved localization accuracy, they rely on assumptions about source models and head geometry that may not hold across individuals or conditions [45]. Deep brain structures critical for emotional processing, including the amygdala and hippocampus, generate weak signals at the scalp that are often indistinguishable from noise, creating a fundamental blind spot in affective neuroscience applications [145,175,655,656].

The signal-to-noise ratio presents another critical limitation, with EEG signals in the microvolt range competing against various noise sources [2]. Physiological artifacts from eye movements, muscle activity, and cardiac signals can exceed neural signals by orders of magnitude. Environmental electromagnetic interference from power lines, monitors, and wireless devices further degrades signal quality. While sophisticated artifact removal techniques using independent component analysis and regression approaches have been developed, they risk removing genuine neural activity along with artifacts, particularly when artifacts and signals of interest overlap in frequency or spatial distribution [398,657,658].

### 8.2. Methodological Challenges in Signal Processing and Analysis

The preprocessing pipeline introduces multiple decision points that can fundamentally alter results, yet no consensus exists on optimal approaches. Choices regarding reference montages (average reference, linked mastoids, or Laplacian), filtering parameters (high-pass cutoffs ranging from 0.1 to 1 Hz), and artifact rejection criteria (amplitude thresholds, probability-based rejection) can produce divergent findings from identical raw data [15]. The reproducibility crisis in neuroscience is partly attributable to this analytical flexibility, with different laboratories employing distinct preprocessing pipelines that hinder cross-study comparisons [659,660,661,662,663].

Feature extraction presents its challenges, with hundreds of potential metrics spanning time, frequency, and spatial domains [2]. Power spectral density, the most common metric, assumes signal stationarity—an assumption violated during dynamic cognitive and emotional processes. Nonetheless, EEG exhibits quasi-stationarity within short windows (2–12 s depending on brain state), justifying standard windowing practices [664,665]. Time-frequency decompositions using wavelets or short-time Fourier transforms introduce time-frequency uncertainty tradeoffs, where improving temporal resolution necessarily degrades frequency resolution [161]. The choice of frequency band boundaries (e.g., alpha defined as 8–12 Hz versus 8–13 Hz) can substantially impact results, particularly given individual differences in peak frequencies that shift with age, cognitive state, and pathology [23,39,552,573].

The multiple comparisons problem becomes acute when analyzing high-dimensional EEG data across numerous electrodes, frequency bands, and time windows. Traditional correction methods like Bonferroni adjustment may be overly conservative, missing genuine effects, while false discovery rate approaches assume independence among tests—an assumption violated by the spatial and temporal correlations inherent in EEG data. Cluster-based permutation testing and other non-parametric approaches offer solutions but require large sample sizes, often impractical in clinical populations [666,667,668,669].

#### Statistical Power and Sample Size Considerations

The robust linear relationship between frontal–midline theta and working memory load (r = 0.65–0.85 across studies) reflects a large effect size (Cohen’s d ≈ 0.8–1.2 for high vs. low load comparisons). Power analysis indicates that detecting this effect with 80% power at α = 0.05 requires minimum sample sizes of n = 15–20 participants for within-subjects designs, though larger samples (n = 30–40) are recommended to achieve adequate power for individual difference analyses and generalization across populations.

However, sample sizes in reviewed studies range from n = 10 to n = 200+, with a median of approximately n = 25. Small samples (n < 20) risk both Type II errors (failing to detect genuine effects) and inflated effect size estimates due to publication bias and the “winner’s curse.” The small sample problem identified in Section 6.7 becomes particularly acute when: (1) investigating subtle effects in clinical populations with high inter-individual variability, (2) employing multi-electrode analyses requiring multiple comparisons corrections, and (3) developing machine learning models where overfitting to small training sets limits generalization.

Recommendations for Future Research:

- Minimum Sample Size Guidelines: For standard cognitive EEG experiments detecting established large effects, n = 30 minimum per group; for exploratory studies or moderate effects, n = 50–100; for individual differences analyses and machine learning applications, n = 100–500+, depending on complexity and feature dimensionality.
- Multi-Site Collaborations: Pool data across laboratories using standardized protocols (EEG-BIDS format, harmonized preprocessing) to achieve adequate power for robust biomarker identification and clinical validation.
- Pre-Registration and Bayesian Approaches: Combat publication bias through pre-registration specifying planned sample sizes, analyses, and stopping rules. Bayesian methods allow sequential designs that stop data collection when sufficient evidence accumulates, balancing efficiency with rigor.
- Effect Size Reporting: Always report effect sizes (Cohen’s d, partial η^2^, correlation coefficients) with confidence intervals, not just significance levels, enabling meta-analyses that aggregate evidence across studies.

### 8.3. Theoretical Gaps in Affective and Cognitive Modeling

The mapping between EEG metrics and psychological constructs suffers from fundamental theoretical ambiguities. The relationship between neural oscillations and subjective experience remains poorly understood, with correlational findings unable to establish causal mechanisms [2]. A particular oscillatory pattern might represent the neural implementation of a cognitive process, a prerequisite enabling condition, an epiphenomenon, or a compensatory response—distinctions that correlational studies cannot resolve [21,233,670,671,672,673,674].

The proliferation of affective models—discrete emotions, dimensional approaches, appraisal theories, and constructionist accounts—creates interpretive challenges when identical EEG patterns receive different labels across frameworks [15]. Frontal alpha asymmetry, extensively studied as a marker of approach–withdrawal motivation, shows inconsistent relationships with emotional valence across studies, suggesting that simplistic one-to-one mappings between neural metrics and affective constructs are inadequate [245]. The cultural specificity of emotional categories further complicates universal mapping attempts, as neural signatures of emotions may vary across populations with different conceptual frameworks for understanding affective experience [238,239,675,676].

Cognitive models face similar challenges, with debates about the fundamental architecture of cognition unresolved. The distinction between automatic and controlled processes, central to dual-process theories, finds inconsistent support in EEG data, with supposedly automatic processes sometimes showing signatures traditionally associated with cognitive control [310]. Working memory models proposing distinct storage buffers for different information types struggle to account for EEG evidence of distributed, overlapping representations that challenge modular views of cognitive architecture [27,677,678,679,680,681].

### 8.4. Individual Differences and Generalizability Constraints

Inter-individual variability in EEG signatures poses fundamental challenges for developing generalizable models. Alpha peak frequency varies from 7 to 14 Hz across healthy adults, with systematic differences related to age, intelligence, and brain volume [23]. What represents optimal brain function for one individual may indicate dysfunction in another—a phenomenon particularly evident in aging research, where maintained high-frequency activity might reflect successful compensation in some individuals but inefficient processing in others [9,64,682,683,684,685].

Baseline differences in skull thickness, scalp properties, and cortical folding patterns affect signal propagation differently across individuals, making standardized montages and analysis approaches suboptimal for capturing individual brain dynamics [175]. The reference electrode problem becomes particularly acute when comparing across individuals with different head geometries, as the same reference location may be differentially influenced by distinct source configurations [82,686,687,688,689,690].

State-dependent variations further complicate interpretation, with factors like time of day, caffeine intake, sleep quality, stress levels, and hormonal fluctuations substantially affecting EEG patterns [143]. The menstrual cycle alone can shift alpha peak frequency by up to 1 Hz, alter inter-hemispheric coherence patterns, and modify emotional reactivity—variations rarely controlled in affective neuroscience studies. These state effects interact with trait differences, making it difficult to separate stable individual characteristics from transient influences [44,690,691,692,693,694].

### 8.5. Integration Challenges Between Affective and Cognitive Domains

The artificial separation between emotion and cognition in research designs fails to capture their fundamental integration in real-world mental processes (Esther) [359]. Laboratory tasks designed to isolate specific cognitive functions or emotional states create artificial situations that may not generalize to naturalistic settings where affective and cognitive processes continuously interact. The ecological validity problem becomes particularly acute when attempting to map findings from controlled experiments using simple stimuli to complex real-world behaviors involving multiple, simultaneous mental processes [341,695,696,697,698,699].

Temporal dynamics pose additional integration challenges, with affective and cognitive processes operating on different timescales [2]. Emotional responses can emerge within 100–200 milliseconds, before conscious awareness, while cognitive evaluation and regulation unfold over seconds. EEG’s excellent temporal resolution captures these dynamics but struggles to disentangle overlapping processes when emotional reactions trigger cognitive responses that in turn modulate ongoing affect [32]. The reciprocal causation between emotion and cognition creates interpretive ambiguities about whether observed neural patterns reflect affective states, cognitive processes, or their interaction [1,510,700,701,702,703].

Cross-frequency coupling mechanisms linking slow and fast oscillations suggest hierarchical organization of brain function, but the functional significance of these interactions for emotion-cognition integration remains unclear [45]. Phase-amplitude coupling between frontal theta and parietal gamma might reflect cognitive control over emotional processes, emotional modulation of attention, or emergent properties irreducible to either domain—interpretations that current analytical approaches cannot definitively distinguish [33,408,435,704,705,706].

### 8.6. Limitations in Clinical Translation and Application

The translation of EEG findings from research settings to clinical applications faces substantial obstacles. Laboratory-grade systems with 64–256 channels, extensive preparation time, and controlled environments differ dramatically from clinical realities requiring rapid assessment with limited channels in noisy environments [138]. Consumer-grade devices promising convenient brain monitoring often sacrifice signal quality for usability, with validation studies showing poor correspondence between their measurements and research-grade systems [10,707,708,709,710].

Diagnostic applications suffer from overlap in EEG signatures across conditions—slowed alpha, increased theta, and reduced coherence characterize numerous neuropsychiatric disorders from depression to dementia, limiting differential diagnostic utility [62,65]. The heterogeneity within diagnostic categories means that group-level differences may not apply to individual patients, with substantial overlap in EEG metrics between clinical and control populations reducing sensitivity and specificity for individual diagnosis [63,711,712].

Intervention applications like neurofeedback show promise but lack standardized protocols, with optimal training parameters (frequency bands, electrode locations, reward thresholds, session numbers) varying across studies [70]. The mechanisms underlying neurofeedback effects remain controversial, with debates about whether improvements reflect specific neural changes or non-specific factors like motivation, attention, and expectancy. The persistence of training effects and their transfer to real-world functioning require longitudinal studies largely absent from current literature [713,714,715,716].

### 8.7. Computational and Statistical Limitations

The curse of dimensionality affects EEG analysis, with typical datasets containing more features than observations [15]. A single EEG recording might generate thousands of potential features across electrodes, frequencies, and time points, but sample sizes rarely exceed hundreds of participants. This dimensionality problem makes machine learning approaches prone to overfitting, with impressive classification accuracies in training data failing to generalize to new samples [717,718,719].

Deep learning approaches achieving near-perfect emotion classification often lack interpretability, functioning as black boxes that provide little insight into underlying mechanisms [20]. The features learned by neural networks may not correspond to neurophysiologically meaningful patterns, limiting their utility for advancing theoretical understanding. Attempts to develop interpretable models often sacrifice performance, creating tensions between prediction accuracy and scientific insight [2,465,720,721].

Small sample sizes endemic to EEG research, particularly in clinical populations, limit statistical power to detect subtle effects and interactions [22]. Effect sizes from underpowered studies are likely inflated, contributing to failures to replicate findings across laboratories. Meta-analyses attempting to synthesize findings face challenges from heterogeneous methodologies, with differences in recording parameters, preprocessing pipelines, and analytical approaches preventing meaningful quantitative synthesis [398,722,723,724,725,726,727].

### 8.8. Ethical and Practical Constraints

The increasing capability to decode mental states from EEG raises ethical concerns about mental privacy, particularly as consumer devices make brain monitoring ubiquitous [138]. The potential for EEG-based discrimination in employment, insurance, or legal contexts requires careful consideration of data protection and use limitations. Current frameworks for informed consent may be inadequate when participants cannot fully understand what information might be extractable from their neural data using future analytical techniques [231,728,729,730,731].

Practical constraints limit the large-scale deployment of EEG-based systems. Electrode preparation and placement require trained personnel, with setup times of 30–60 min for research-grade systems [2]. Gel-based electrodes provide better signal quality but are messy and uncomfortable for extended wear. Dry electrode alternatives sacrifice signal quality for convenience, with higher impedances and greater susceptibility to motion artifacts [10]. Wireless systems introduce additional challenges from battery limitations, data transmission reliability, and synchronization with other devices [138,732,733,734,735].

Subject identification achieving up to 98% accuracy using spectral power features raises particular privacy concerns, as EEG patterns may serve as biometric identifiers that cannot be changed if compromised [15]. The persistence of individual EEG signatures across sessions and conditions suggests that anonymization of neural data may be impossible, requiring reconsideration of data sharing practices and privacy protections [731,736,737].

### 8.9. Future Challenges in Advancing the Field

Addressing these multifaceted challenges requires coordinated efforts across technical, theoretical, and practical domains. Technical advances in hardware (higher-density arrays, active electrodes, improved amplifiers) must be matched by theoretical sophistication in linking neural dynamics to psychological constructs [2]. The development of standardized preprocessing pipelines and feature extraction methods could improve reproducibility, but must balance standardization with flexibility to accommodate legitimate methodological diversity [15,74,738,739,740,741,742,743,744].

Large-scale collaborative projects generating open datasets with diverse populations, standardized protocols, and comprehensive phenotyping could address sample size limitations and individual difference challenges. However, such initiatives require substantial resources and coordination across institutions, countries, and research traditions with different priorities and approaches [745,746,747,748,749,750].

The integration of EEG with other neuroimaging modalities (concurrent EEG-fMRI), peripheral physiological measures, and behavioral assessments promises more comprehensive characterization of brain-behavior relationships but introduces additional complexity in data acquisition, analysis, and interpretation [341]. Real-time processing capabilities enabling closed-loop applications must overcome computational constraints while maintaining scientific rigor in rapidly evolving neural states [2,262,644,751,752,753,754,755].

### 8.10. Summary of Key Limitations

The challenges facing EEG-based modeling of affective and cognitive processes are interconnected and mutually reinforcing. Technical limitations in spatial resolution interact with theoretical ambiguities about the neural implementation of psychological constructs [138,175]. Individual differences complicate group-level analyses, while group-level findings may not apply to individuals [23]. The separation of emotion and cognition in research designs fails to capture their integration in naturalistic behavior (Esther) [1,359].

These challenges are not merely technical obstacles to be overcome through better equipment or analytical methods but reflect fundamental questions about the relationship between brain activity and mental life that require conceptual as well as methodological innovation [2]. The contradictory results often observed across studies, where different models might assign different labels to the same signal, further complicate interpretation [398].

Despite these limitations, EEG remains an invaluable tool for investigating brain function, particularly when its constraints are explicitly acknowledged and addressed through careful experimental design, appropriate analytical approaches, and cautious interpretation [15]. The path forward requires not minimizing these challenges but confronting them directly through convergent evidence across methods, levels of analysis, and theoretical frameworks. Only through honest recognition of current limitations can the field advance toward more complete understanding of how neural oscillations give rise to the rich complexity of human cognition and emotion [2].

**Figure 7:**
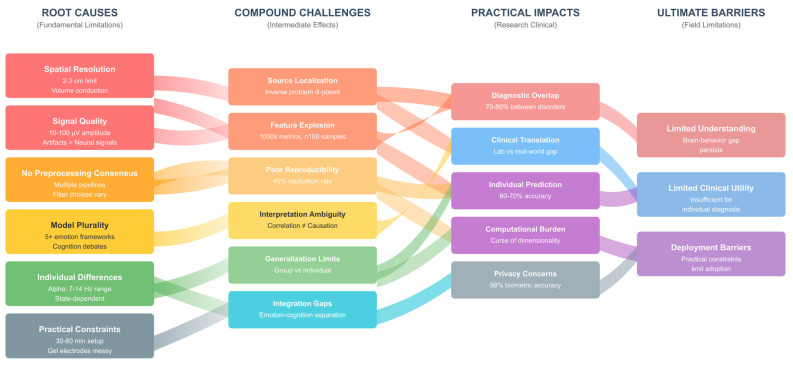
Challenges in EEG-based cognitive and affective mapping.

The visualization below (Figure 7) traces how fundamental limitations (left column) flow through compound challenges (middle-left) and practical impacts (middle-right) to create ultimate barriers (right column) that limit the field’s advancement.

Root causes include technical constraints (spatial resolution 2–3 cm, signal quality 10–100 μV), methodological issues (no preprocessing consensus), theoretical gaps (5+ competing emotion frameworks), individual variability (alpha frequency 7–14 Hz range), and practical constraints (30–60 min setup). These fundamental limitations generate compound challenges such as the ill-posed inverse problem, feature explosion (1000 s of metrics vs. <100 samples), poor reproducibility (<40% replication rate), interpretation ambiguity, and integration gaps between emotion and cognition. The cascading effects converge at practical impacts, including diagnostic overlap (70–80% between disorders), limited clinical translation, poor individual prediction accuracy (60–70%), computational burden, and privacy concerns (98% biometric identification). Ultimately, these challenges culminate in three major barriers: limited understanding of brain-behavior relationships, insufficient clinical utility for individual diagnosis, and practical deployment constraints. Flow width represents impact magnitude, while color gradients (red: technical, orange: methodological, yellow: theoretical, green: individual, gray: practical) distinguish challenge categories. The diagram emphasizes that addressing root causes on the left would be more effective than treating downstream symptoms, highlighting critical intervention points for advancing the field.

## 9. Future Directions—Technological Advances and Emerging Applications

### 9.1. Next-Generation EEG Technologies

The evolution of EEG technology continues to accelerate, driven by advances in materials science, signal processing, and miniaturization. Building on the foundations described in Section 2, next-generation systems are addressing fundamental limitations while opening new research paradigms [2,14,138,756].

The transition from traditional wired systems to wireless, high-density configurations represents a paradigm shift in EEG acquisition. As noted by the study [10], systems like the Emotiv Epoc neuroheadset, with its 14 electrodes and 128 Hz sampling rate, demonstrate that consumer-grade devices can capture meaningful neural signals without conductive gel, using only sterile saline solution. Future developments point toward dry electrode technologies with 256+ channels that maintain signal quality comparable to wet electrodes while enabling prolonged recordings in naturalistic settings [138,707,757,758,759,760].

Advanced materials, including graphene-based electrodes and flexible electronics, promise to overcome the comfort and usability limitations that have confined EEG primarily to laboratory settings. These innovations enable continuous 24 h monitoring, crucial for understanding circadian variations in cognitive and affective states and for capturing rare clinical events in neurological conditions [175]. The rapid progress in consumer-grade wearable EEG devices, while accompanied by concerns about feasibility and scientific adequacy, opens unprecedented opportunities for ecological validity in neuroscience research [138,761,762,763,764,765].

The integration of EEG with complementary neuroimaging modalities addresses the inherent trade-offs between spatial and temporal resolution. As researchers in their study [231] demonstrate, simultaneous EEG-fMRI recordings, once plagued by artifacts, now benefit from sophisticated removal algorithms that preserve neural signals while eliminating scanner-induced noise. This multimodal approach enables researchers to link millisecond-scale oscillatory dynamics to hemodynamic responses, bridging multiple scales of brain organization [193,196,197,341,766,767].

Emerging combinations include EEG with near-infrared spectroscopy (NIRS) for portable brain-behavior monitoring, and integration with magnetoencephalography (MEG) for enhanced source localization [45]. These hybrid approaches are particularly valuable for mapping the interplay between emotion and cognition discussed in Section 7, where distributed networks operate across multiple spatial and temporal scales [15,262,754,768,769].

### 9.2. Advanced Analytical Frameworks

The application of artificial intelligence to EEG analysis has progressed from simple classifiers to sophisticated deep learning architectures that can identify subtle patterns invisible to traditional analyses. As highlighted by researchers, emotion recognition systems now achieve accuracy rates exceeding 98% for subject identification and 85–90% for affective state classification using combinations of spectral features, connectivity measures, and nonlinear dynamics [15,272,273,274,770,771].

Future developments focus on interpretable AI models that not only classify mental states but also reveal the underlying neural mechanisms. As the researcher in the study [20] demonstrates, attention-based neural networks can identify which frequency bands and electrode locations contribute most to classification decisions, providing insights that inform theoretical models. Transfer learning approaches enable models trained on large datasets to adapt to individual users with minimal calibration, addressing the challenge of inter-individual variability emphasized by the studies [23,772,773,774,775,776].

The brain operates as a complex network, with cognitive and affective processes emerging from dynamic interactions between distributed regions. Graph theoretical approaches quantify network properties including clustering, path length, and hub organization, revealing how network topology relates to cognitive performance and emotional regulation [21]. As demonstrated by the study [22], microstate analysis reveals quasi-stable topographical patterns lasting 80–120 milliseconds that represent the building blocks of mental processes [53,777,778,779,780,781].

Future applications will leverage dynamic network analysis to track how brain networks reconfigure in response to cognitive demands and emotional challenges [45]. These approaches are particularly relevant for understanding the integration of affective and cognitive processes, where the same neural substrates support both emotional and executive functions (Esther) [359]. Multilayer network models that incorporate multiple frequency bands simultaneously promise to capture cross-frequency interactions crucial for binding distributed information into coherent mental states [44,782,783,784,785,786].

### 9.3. Clinical Translation and Biomarker Development

The heterogeneity of neuropsychiatric conditions demands personalized approaches that account for individual differences in neural function. EEG biomarkers offer objective measures that can guide treatment selection and monitor therapeutic response [1]. In depression, frontal alpha asymmetry and theta cordance predict antidepressant response [33], while gamma-band abnormalities in schizophrenia correlate with symptom severity and cognitive deficits [62,63,787,788,789,790].

Future developments will establish normative databases stratified by age, sex, and other demographic factors, enabling clinicians to identify deviations indicative of emerging pathology [23]. Machine learning models trained on longitudinal data will predict disease trajectories and identify optimal intervention windows [9]. The integration of EEG with genetic, metabolic, and behavioral data within precision medicine frameworks promises to revolutionize psychiatric diagnosis and treatment [64,65,791,792,793,794,795,796].

Real-time EEG processing enables closed-loop interventions that modulate brain activity to achieve therapeutic goals. Neurofeedback protocols targeting specific frequency bands show efficacy for attention disorders, anxiety, and cognitive enhancement [70]. Future systems will employ machine learning to optimize feedback parameters dynamically, adapting to individual learning curves and maximizing therapeutic outcomes [2,71,401,714,797,798].

Non-invasive brain stimulation techniques, including transcranial magnetic stimulation (TMS) and transcranial electrical stimulation (tES), benefit from EEG guidance to target specific networks and monitor immediate effects [143]. Closed-loop stimulation systems that adjust parameters based on ongoing brain states promise enhanced efficacy with reduced side effects. These approaches are particularly relevant for cognitive enhancement applications, where stimulation parameters can be optimized to facilitate specific cognitive processes identified through established mappings [82,799,800,801,802,803].

### 9.4. Affective Computing and Human–Computer Interaction

The mappings between EEG metrics and affective models enable the development of systems that respond to users’ emotional states in real time [15]. Applications span from educational technologies that adapt content difficulty based on engagement and frustration levels to therapeutic virtual reality environments that modulate scenarios according to anxiety levels [2]. As the study [161] demonstrates, analyzing oscillations across standard frequency bands and systematization of EEG activity provides robust emotion recognition capabilities [18,655,804,805,806].

Future emotion-aware systems will integrate multiple physiological signals with contextual information to achieve robust emotion recognition across diverse populations and situations [138]. Federated learning approaches will enable models to improve continuously while preserving user privacy, addressing the ethical concerns raised regarding brain data. The challenge lies in developing systems that not only detect emotions accurately but also respond appropriately to support user wellbeing [245,807,808,809,810,811].

For individuals with severe motor disabilities, EEG-based brain–computer interfaces (BCIs) offer communication channels independent of muscle control. Current P300 spellers and motor imagery systems achieve information transfer rates of 20–40 bits per minute [310]. Future developments focus on hybrid BCIs that combine multiple control signals—P300, steady-state visual evoked potentials (SSVEPs), and motor imagery—to improve speed and reliability [812,813,814,815,816,817].

Advances in decoder algorithms, particularly deep learning approaches that adapt to non-stationary signals, promise to increase communication rates while reducing user fatigue. The integration of natural language processing with BCI systems will enable more intuitive communication, moving beyond character selection to word and phrase prediction based on neural patterns associated with language planning [818,819,820,821].

### 9.5. Cognitive Enhancement and Optimization

The cognitive models and their EEG correlates inform the development of targeted training protocols that enhance specific cognitive abilities. Adaptive training systems that adjust difficulty based on real-time cognitive load measurements optimize the challenge level to maintain engagement without overwhelming users [27,601,822,823,824,825].

Future cognitive training platforms will leverage EEG markers of learning and consolidation to personalize training schedules and identify optimal learning states [32]. The combination of cognitive training with neurostimulation, guided by EEG biomarkers, shows promise for accelerating skill acquisition and enhancing transfer to untrained tasks [70]. These approaches have implications for education, professional training, and rehabilitation following brain injury [822,826,827,828].

Portable EEG systems enable monitoring of cognitive states during real-world activities, from driving and aviation to surgical procedures and military operations. Fatigue detection systems based on theta/beta ratios and alpha spindles can prevent accidents by alerting operators or triggering automated safety systems [39,829,830,831,832,833].

Future applications will integrate EEG with environmental sensors and behavioral data to create comprehensive models of human performance in complex environments [398]. Machine learning algorithms will predict performance decrements before they manifest behaviorally, enabling proactive interventions. The challenge lies in developing robust algorithms that generalize across individuals and situations while maintaining sensitivity to subtle changes in cognitive state [23,572,834,835,836,837,838].

### 9.6. Theoretical Advances and Integrative Models

The evidence for deep integration between affective and cognitive processes necessitates theoretical frameworks that transcend traditional boundaries. As (Esther) researchers in the study [359] emphasize, the effect of emotions on attention, decision-making, and memory has attracted widespread scientific interest, yet mechanisms remain ambiguous. Future models will characterize mental states along multiple dimensions simultaneously, recognizing that emotions are cognitive processes and that cognition is inherently affective [2,839,840,841,842,843,844].

Computational models that simulate the emergence of affective–cognitive states from neural dynamics will bridge levels of analysis from cellular mechanisms to behavior [15]. These models will incorporate principles from dynamical systems theory, predictive coding, and embodied cognition to explain how brain, body, and environment interact to generate conscious experience [245,845,846,847,848,849].

The substantial inter-individual variability in EEG signatures demands frameworks that account for individual differences in brain structure, function, and experience [23]. Future research will identify neurocognitive phenotypes that explain why individuals differ in their emotional reactivity, cognitive abilities, and vulnerability to mental illness [9,25,26,358,461,850].

Longitudinal studies tracking individuals across development and aging will reveal how EEG signatures evolve and how early patterns predict later outcomes [65]. Machine learning approaches that learn personalized models from limited data will enable precision applications while respecting individual uniqueness [20]. These developments have implications for education, where teaching methods can be tailored to individual learning styles, and for clinical practice, where treatments can be matched to individual neurocognitive profiles [64,397,711,851,852,853,854].

### 9.7. Emerging Application Domains

The COVID-19 pandemic accelerated the adoption of digital health technologies, including remote EEG monitoring for mental health assessment. Future platforms will enable individuals to track their cognitive and emotional states at home, with AI systems providing personalized insights and recommendations [2,8,855,856,857,858].

Integration with smartphone sensors and wearable devices will create comprehensive digital phenotypes that capture multiple aspects of mental health [10]. Machine learning models will identify early warning signs of relapse in mood disorders, cognitive decline in neurodegenerative diseases, and treatment response in various conditions (63. The challenge lies in developing systems that are clinically valid, user-friendly, and respectful of privacy and autonomy [138,859,860,861,862,863,864].

As virtual and augmented reality technologies become ubiquitous, EEG-based assessment of user experience becomes crucial for optimizing immersive environments. Neural markers of presence, engagement, and cognitive load will guide the design of virtual worlds that are both compelling and cognitively sustainable [2,815,865,866,867].

Future AR/VR systems will adapt in real-time to users’ mental states, modulating stimulus intensity, complexity, and emotional valence to maintain optimal engagement [161]. Therapeutic applications will leverage these capabilities to create personalized exposure therapies for anxiety disorders, immersive cognitive training for dementia, and social skills training for autism spectrum disorders [70,868,869,870,871,872].

### 9.8. Methodological Innovations

The methodological approaches increasingly emphasize ecological validity through naturalistic paradigms. Future studies will employ mobile EEG during real-world activities, from classroom learning to social interactions, capturing the complexity of cognition and emotion in everyday life [3,138,231,639,873,874,875].

Hyperscanning techniques that record EEG simultaneously from multiple individuals will reveal the neural basis of social cognition, empathy, and interpersonal synchrony [341]. These approaches are particularly relevant for understanding disorders characterized by social deficits and for developing interventions that target social brain networks [62,876,877,878,879].

The complexity of brain-behavior relationships demands large-scale collaborative efforts that aggregate data across laboratories and populations. Future initiatives will establish standardized protocols for data collection, preprocessing, and analysis, enabling meta-analyses that identify robust biomarkers and resolve conflicting findings [1,2,880,881,882,883].

Cloud-based platforms will enable researchers to share data and algorithms while maintaining privacy through federated learning and differential privacy techniques. These collaborative approaches will accelerate discovery while ensuring that findings generalize across diverse populations and settings [23,884,885,886,887,888,889].

### 9.9. Convergence with Other Technologies

Understanding how genetic variations influence EEG patterns and their relationship to cognition and emotion represents a frontier in neuroscience. Genome-wide association studies are identifying genetic variants associated with EEG features, while proteomic analyses reveal molecular mechanisms underlying oscillatory dynamics [175,890,891,892,893,894,895,896,897,898].

Future research will integrate multi-omic data with EEG to create comprehensive models of brain function that span from molecules to behavior [45]. These approaches will identify novel therapeutic targets and enable prediction of treatment response based on integrated biomarkers. The convergence of neurotechnology with precision medicine promises to transform the diagnosis and treatment of brain disorders [64,65,899,900,901,902,903].

The computational demands of analyzing high-dimensional EEG data and simulating brain dynamics push the limits of classical computing. Quantum computing offers potential solutions for optimization problems in source localization, network analysis, and pattern recognition [21,904,905,906,907,908,909,910].

Future applications will leverage quantum algorithms for feature selection, classification, and simulation of quantum processes potentially relevant to consciousness [22]. While practical quantum computers remain limited, hybrid classical-quantum approaches show promise for specific neuroscience applications [20,911,912,913,914].

### 9.10. Synthesis and Vision for the Future

The future of EEG-based cognitive and affective neuroscience lies in the convergence of technological innovation, theoretical sophistication, and practical application. The mappings between EEG metrics and psychological models established throughout this review provide the foundation for a new era of brain-based technologies that enhance human capabilities while respecting human values [2,15].

Key priorities for advancing the field include the following:

1. Developing robust, generalizable biomarkers that account for individual differences while maintaining diagnostic and prognostic value [9,23];
2. Creating interpretable AI models that not only classify brain states but also reveal underlying mechanisms [20];
3. Establishing ethical frameworks that protect individual privacy and autonomy while enabling beneficial applications [138,231];
4. Building inclusive technologies that work across diverse populations and address health disparities [8];
5. Fostering interdisciplinary collaboration that integrates neuroscience, psychology, engineering, and clinical practice [1,2].

The ultimate vision is of a future where understanding of brain-behavior relationships enables technologies that augment human cognition, facilitate emotional well-being, and treat neuropsychiatric conditions with precision and compassion. As emphasized by the study [2], the opportunities and advantages derived from these implementations underscore the potential of the proposed mappings, which provide convenient insights into the development of a diverse range of applications and experimental settings [751,915,916,917]. The comprehensive EEG mappings appear particularly appropriate in responsive or adaptive applications as modulation signals, especially when emotional and cognitive features must be modeled in human-centric adaptive systems [15]. Achieving this vision requires continued investment in basic research, thoughtful consideration of ethical implications, and commitment to translating discoveries into accessible, beneficial applications for all members of society [58,918,919,920].

As we stand at the threshold of the neurotechnology revolution, the comprehensive frameworks linking EEG metrics to human affective and cognitive models presented in this review provide essential guidance for navigating the opportunities and challenges ahead. The journey from detecting electrical brain activity to understanding and enhancing human mental life represents one of science’s grand challenges—one that promises to transform our understanding of ourselves and our potential as a species [1,2].

**Figure 8:**
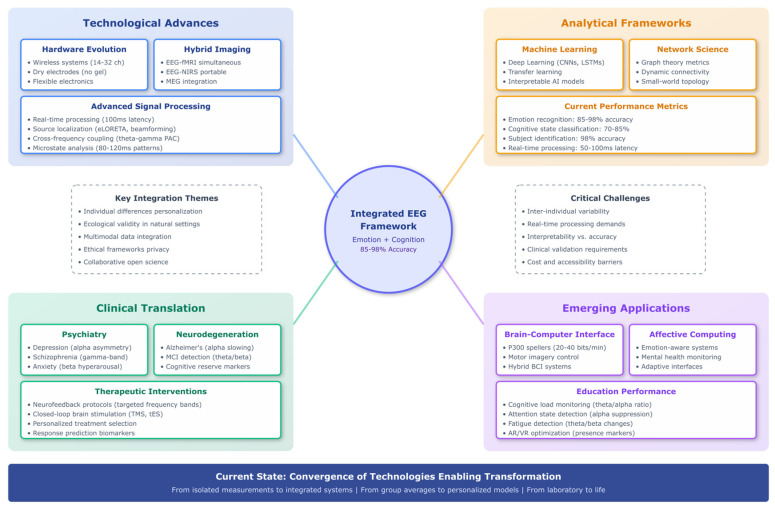
Convergent technologies and applications in EEG-based neuroscience.

Figure 8 synthesizes the convergent technologies and applications discussed throughout this section into a four-quadrant framework centered on an integrated EEG system achieving 85–98% emotion recognition accuracy. The visualization captures the current state of the field rather than projecting future scenarios, organizing existing capabilities and near-term developments across technological advances (wireless systems, dry electrodes, hybrid imaging), analytical frameworks (deep learning, network science, interpretable AI), clinical translation (psychiatric biomarkers, neurofeedback, brain stimulation), and emerging applications (BCIs, affective computing, educational technology). Each quadrant contains specific technologies and metrics drawn from the reviewed literature, with connecting lines to the central hub emphasizing the interdependence of these domains. The inclusion of key integration themes (personalization, ecological validity, multimodal data) and critical challenges (inter-individual variability, real-time processing, clinical validation) reflects the balanced perspective maintained throughout Section 9. This grounded visualization demonstrates that the transformation of EEG from a measurement tool to an integrated neurotechnology platform is not a distant vision but an ongoing convergence of multiple technological and methodological advances, each contributing essential capabilities to the unified framework that promises to enhance understanding and support of human cognitive and emotional processes.

## 10. Ethical Considerations in EEG-Based Brain-Reading Technologies

The capacity of modern EEG systems to decode mental states with unprecedented accuracy fundamentally challenges traditional concepts of privacy. As the study [15] demonstrated, spectral power analysis across delta (1–4 Hz), theta (4–8 Hz), alpha (8–12 Hz), beta (12–30 Hz), and gamma (30–50 Hz) frequency bands can identify individual subjects with up to 98% accuracy from a library of 64 participants. This biometric capability extends beyond identification to reveal cognitive and emotional states that individuals may not consciously choose to disclose [138].

The sensitivity of neural data exceeds conventional physiological measures because EEG directly records activity from the organ underlying decision-making, language, and memory processes [138]. Machine learning pipelines combining linear features such as hemispheric asymmetry with nonlinear multiscale entropy measures can assess emotional states with 10–20% better accuracy than participants’ own self-reports [138]. This suggests EEG systems may detect subconscious responses, emotional vulnerabilities, or cognitive characteristics without explicit awareness or consent.

Pattern analyses of EEG recordings can potentially reveal personal attributes, including sexual orientation, religious beliefs, political preferences, and mental health status, through indirect inference [231]. Unlike passwords or other changeable identifiers, neural signatures represent immutable biological characteristics that, if compromised, cannot be reset or replaced. The retroactive risk posed by archived EEG data is particularly concerning, as future analytical advances may extract information not currently accessible [2].

Traditional informed consent frameworks prove inadequate for EEG research, given the complexity and evolving nature of neural data interpretation. As researchers in their study [231] emphasize, researchers must recognize that tracking devices and behavioral pattern analyses can reveal personal details without explicit participant consent, necessitating full disclosure of indirect information gathering potential. The multidimensional nature of EEG signals means data collected for specific research purposes could yield unrelated insights about cognitive abilities, emotional regulation, or neurological conditions [15].

Special protective measures are essential when collecting EEG data from vulnerable populations. Children’s developing neural patterns may not predict adult characteristics, yet childhood EEG profiles could impact future educational or employment opportunities if accessed inappropriately [2]. For elderly individuals with cognitive decline or persons with psychiatric conditions exhibiting fluctuating capacity, consent procedures must accommodate varying comprehension levels across different states [138].

The integration of EEG monitoring in educational settings using affordable devices like the Emotiv EPOC—featuring 14 electrodes with 128 Hz sampling rate and requiring only saline solution rather than conductive gel—raises complex questions about parental consent, child assent, and long-term implications of neural profiling during development [10]. Researchers should obtain informed consent from each participant prior to experimental procedures while ensuring participants understand potential uses of data beyond immediate research objectives [231].

The proliferation of portable EEG systems enabling continuous monitoring outside laboratory settings creates opportunities for beneficial applications while introducing risks of surveillance and discrimination. Virtual environments can leverage EEG-based emotion recognition to enhance interaction transparency without requiring user training, with applications spanning augmented reality, medicine, education, entertainment, and lifestyle domains [2]. However, these same capabilities enable workplace monitoring that could exclude individuals based on neural patterns rather than performance, or educational categorization that creates self-fulfilling prophecies.

EEG-based affective computing applications must carefully balance potential benefits against privacy risks. While emotion modulation in clinical, educational, and commercial environments offers therapeutic value [2], the ability to detect emotional states more accurately than self-report raises concerns about mental autonomy and authentic self-expression. The development of consumer neurotechnologies for meditation, focus enhancement, or emotional regulation bypasses traditional research oversight while collecting sensitive neural data from users who may not comprehend the implications [10].

The dual-use nature of EEG technologies presents particular challenges. Systems developed for medical diagnosis or cognitive assessment could be repurposed for security screening, deception detection, or surveillance applications that threaten fundamental rights to mental privacy and presumption of innocence [138]. The variable accuracy of EEG-based inference across individuals and contexts, combined with the black-box nature of many machine learning algorithms, makes such applications especially problematic for high-stakes decisions affecting liberty and justice [15].

Responsible development of EEG-based brain-reading technologies requires comprehensive governance frameworks that extend beyond existing medical device regulations and research ethics guidelines. Researchers in their study [138] argue that ethical considerations for EEG applications augmenting or influencing brain-based functions are at least as fundamental as those in medical domains, yet current regulatory structures inadequately address the unique challenges of continuous neural monitoring and mental state inference.

Essential governance principles must include strict purpose limitation preventing function creep, transparency requirements for algorithms and inferences, user control over data collection and analysis, and equitable benefit sharing from population-level research insights [2]. All communications arising from EEG experiments should clearly emphasize the nature and intended application of collected data and derived results [138].

Researchers must implement privacy-by-design principles, develop clear data management plans addressing long-term storage and secondary use, and establish community advisory boards including diverse stakeholders [231]. Technology developers should conduct ethical impact assessments, implement end-to-end encryption, provide meaningful user control, and engage neuroethics experts throughout the development process [10]. The field requires interdisciplinary collaboration among neuroscientists, ethicists, legal scholars, policymakers, and affected communities to identify emerging issues and develop solutions balancing innovation with protection of neural privacy [2].

## 11. Conclusions

This interdisciplinary scoping review demonstrates that EEG-based mapping of affective and cognitive states has achieved significant empirical validation, with emotion recognition accuracies reaching 85–98% and cognitive state classification achieving 70–95% accuracy using advanced machine learning approaches. The convergence of evidence across 95+ studies establishes robust associations between frequency-specific oscillations and psychological constructs: frontal alpha asymmetry (8–13 Hz) as a reliable marker of emotional valence, frontal–midline theta (4–8 Hz) as an index of cognitive load and working memory demands, and cross-frequency coupling mechanisms as coordinators of integrated affective–cognitive processing.

Despite these advances, fundamental challenges constrain clinical translation and real-world deployment. Technical limitations—including spatial resolution constraints (2–3 cm), susceptibility to artifacts (SNR: 10–100 μV), and the mathematically ill-posed inverse problem—restrict precise source localization. Methodological inconsistencies across laboratories, with no consensus on preprocessing pipelines or frequency band definitions, compromise reproducibility and meta-analytic synthesis. Inter-individual variability remains substantial, with alpha peak frequency ranging from 7–14 Hz across healthy adults and further modulated by age, cognitive reserve, and pathology. The overlap of EEG signatures across neuropsychiatric conditions (70–80% similarity between disorders) limits differential diagnostic utility, while individual prediction accuracy lags behind group-level discrimination.

The evidence overwhelmingly supports an integrated view of affective–cognitive processing, challenging traditional dichotomies. Neural oscillations serve multiple functions through flexible network reconfiguration rather than dedicated emotional or cognitive modules. This integration manifests through theta-gamma coupling in frontal regions for emotion-regulated working memory, alpha–beta interactions for affective gating of attention, and distributed network dynamics that adapt to concurrent emotional and cognitive demands. These findings necessitate theoretical frameworks that accommodate the fundamental intertwining of feeling and thinking in adaptive behavior.

Future progress requires coordinated advances across multiple domains: (1) technological innovations including high-density wireless systems and hybrid neuroimaging approaches to overcome current limitations; (2) standardized protocols and large-scale collaborative databases to address reproducibility and generalizability; (3) interpretable AI models that reveal mechanisms rather than merely optimize classification; (4) personalized approaches accounting for individual neural signatures and trajectories; and (5) robust ethical frameworks protecting mental privacy while enabling beneficial applications. The path from laboratory demonstrations to clinical implementation demands not only technical refinement but also theoretical sophistication in understanding how 10^9 neurons generate the rich complexity of human mental life through rhythmic electrical activity measurable at the scalp. As the field stands at the threshold of widespread neurotechnology deployment, this review’s integrated framework provides essential guidance for realizing EEG’s potential to enhance understanding and support of human cognitive and emotional processes while respecting fundamental human values.

## Figures and Tables

**Table 1:** Standardized EEG frequency-band definitions used throughout this review.

Band	Frequency Range	Primary Functional Associations	Key Neural Mechanisms
Delta (δ)	0.5–4 Hz	Deep sleep, unconsciousness, pathological states, motivational salience	Cortical–thalamic loops, slow-wave sleep generation
Theta (θ)	4–8 Hz	Memory encoding, cognitive control, drowsiness, emotional processing	Hippocampal–cortical dialogue, working memory maintenance
Alpha (α)	8–13 Hz	Relaxed wakefulness, attention, cortical inhibition, sensory gating	Thalamo–cortical rhythms, functional inhibition
Beta (β)	13–30 Hz	Active thinking, motor planning, focus, anxiety, cognitive processing	Cortico-cortical communication, motor control
Gamma (γ)	>30 Hz	Perceptual binding, consciousness, attention, sensory processing	Local cortical circuits, feature integration

Note: These represent conventionally defined bands suitable for group-level analyses and cross-study comparisons.

## Data Availability

No new data were created or analyzed in this study.

## Supplementary Materials

The following supporting information can be downloaded at https://www.mdpi.com/article/10.3390/biomimetics10110730/s1, Table S1: Abbreviation list.
