# Supplementary material for: Mapping EEG Metrics to Human Affective and Cognitive Models: An Interdisciplinary Scoping Review from a Cognitive Neuroscience Perspective
Source: Biomimetics (Basel). 2025 Nov 1;10(11):730. doi: 10.3390/biomimetics10110730 (PMC12649996; doi:10.3390/biomimetics10110730)
Supplement: Supplementary file 1 [file biomimetics-10-00730-s001.zip › biomimetics-3851024-supplementary.pdf]

Table S1. Abbreviation List

Neuroimaging Techniques

EEG - Electroencephalography
fMRI - functional Magnetic Resonance Imaging
MEG - Magnetoencephalography
NIRS - Near-Infrared Spectroscopy
TMS - Transcranial Magnetic Stimulation
tES - transcranial Electrical Stimulation
tDCS - transcranial Direct Current Stimulation

EEG-Specific Terms and Analysis Methods

ERP - Event-Related Potential
ERSP - Event-Related Spectral Perturbations
ICA - Independent Component Analysis
LORETA - Low-Resolution Electromagnetic Tomography
eLORETA - exact Low-Resolution Electromagnetic Tomography
sLORETA - standardized Low-Resolution Electromagnetic Tomography
PLV - Phase-Locking Value
wPLI - weighted Phase Lag Index
SSVEP - Steady-State Visual Evoked Potentials
FMθ (FMT) - Frontal Midline Theta
EOG - Electrooculogram
EMG - Electromyogram

ERP Components

P1 - Positive component at ~100ms
N1 - Negative component at ~100-150ms
P300 (P3) - Positive component at ~300ms
P3a - Novelty P3 subcomponent
P3b - Target P3 subcomponent
N200 (N2) - Negative component at ~200ms
EPN - Early Posterior Negativity
LPP - Late Positive Potential
CNV - Contingent Negative Variation
ERN - Error-Related Negativity
FRN - Feedback-Related Negativity

Brain Regions

Fz - Frontal midline electrode position
FCz - Frontocentral electrode position
Cz - Central electrode position
pFC - prefrontal Cortex
vlPFC - ventrolateral Prefrontal Cortex
dlPFC - dorsolateral Prefrontal Cortex
ACC - Anterior Cingulate Cortex
pLOFC - parieto-lateral occipital frontal cortex
pMFC - parietal medial frontal cortex

Clinical and Neuropsychological Terms

ADHD - Attention Deficit Hyperactivity Disorder
MDD - Major Depressive Disorder
PTSD - Post-Traumatic Stress Disorder
AD - Alzheimer's Disease
MCI - Mild Cognitive Impairment
TBI - Traumatic Brain Injury
VAD - Valence-Arousal-Dominance
EF - Executive Function
CLT - Cognitive Load Theory

DMC - Dual Mechanisms of Control
WM - Working Memory

Computational and Technical Terms

AI - Artificial Intelligence
ML - Machine Learning
DL - Deep Learning
CNN - Convolutional Neural Networks
RNN - Recurrent Neural Networks
LSTM - Long Short-Term Memory
SVM - Support Vector Machines
LDA - Linear Discriminant Analysis
RF - Random Forests
BCI - Brain-Computer Interface
FFT - Fast Fourier Transform
PSD - Power Spectral Density
HHT - Hilbert-Huang Transform

Databases and Standards

DEAP - Database for Emotion Analysis using Physiological Signals
IAPS - International Affective Picture System
GAPED - Geneva Affective Picture Database
MAHNOB-HCI - Multimodal Database for Affect Recognition and Implicit Tagging
BIDS - Brain Imaging Data Structure
EEG-BIDS - EEG Brain Imaging Data Structure
10-20 System - International 10-20 System for electrode placement
10-10 System - Extended International 10-10 System

Physiological Measures and Units

Hz - Hertz
kHz - kilohertz
ms - milliseconds
s - seconds
μV - microvolts
mV - millivolts
μs - microseconds
dB - decibels
SNR - Signal-to-Noise Ratio
HRV - Heart Rate Variability
GSR - Galvanic Skin Response
SCR - Skin Conductance Response

Frequency Bands

δ (delta) - 0.5-4 Hz
θ (theta) - 4-8 Hz
α (alpha) - 8-13 Hz
β (beta) - 13-30 Hz
γ (gamma) - 30-80 Hz

Neurofeedback and Intervention Terms

NFB - Neurofeedback
CBT - Cognitive Behavioral Therapy
ECT - Electroconvulsive Therapy
rTMS - repetitive Transcranial Magnetic Stimulation

Molecular and Genetic Terms

5-HTTLPR - Serotonin Transporter Gene-Linked Polymorphic Region
COMT - Catechol-O-Methyltransferase
NMDA - N-Methyl-D-Aspartate
GABA - Gamma-Aminobutyric Acid

Other Technical Terms

AR/VR - Augmented Reality/Virtual Reality
GUI - Graphical User Interface
API - Application Programming Interface
BOLD - Blood Oxygen Level Dependent
ROI - Region of Interest
ISI - Inter-Stimulus Interval
ITI - Inter-Trial Interval
RT - Reaction Time
SOA - Stimulus Onset Asynchrony

Statistical Terms

ANOVA - Analysis of Variance
FDR - False Discovery Rate
FWE - Family-Wise Error
SD - Standard Deviation
SE - Standard Error
CI - Confidence Interval
AUC - Area Under the Curve
ROC - Receiver Operating Characteristic
